# Caloric restriction blocks neuropathology and motor deficits in Machado–Joseph disease mouse models through SIRT1 pathway

**DOI:** 10.1038/ncomms11445

**Published:** 2016-05-11

**Authors:** Janete Cunha-Santos, Joana Duarte-Neves, Vitor Carmona, Leonard Guarente, Luís Pereira de Almeida, Cláudia Cavadas

**Affiliations:** 1 CNC—Center for Neuroscience and Cell Biology, University of Coimbra, Rua Larga, Coimbra 3004-504, Portugal; 2 Faculty of Pharmacy, University of Coimbra, Coimbra 3000-548, Portugal; 3 Department of Biology, Massachusetts Institute of Technology, Cambridge, Massachusetts 02139, USA; 4 Glenn Laboratory for the Science of Aging, Massachusetts Institute of Technology, Cambridge, Massachusetts 02139, USA; 5 Koch Institute for Integrative Cancer Research, Massachusetts Institute of Technology, Cambridge, Massachusetts 02139, USA

## Abstract

Machado–Joseph disease (MJD) is a neurodegenerative disorder characterized by an abnormal expansion of the CAG triplet in the *ATXN3* gene, translating into a polyglutamine tract within the ataxin-3 protein. The available treatments only ameliorate symptomatology and do not block disease progression. In this study we find that caloric restriction dramatically rescues the motor incoordination, imbalance and the associated neuropathology in transgenic MJD mice. We further show that caloric restriction rescues SIRT1 levels in transgenic MJD mice, whereas silencing SIRT1 is sufficient to prevent the beneficial effects on MJD pathology. In addition, the re-establishment of SIRT1 levels in MJD mouse model, through the gene delivery approach, significantly ameliorates neuropathology, reducing neuroinflammation and activating autophagy. Furthermore, the pharmacological activation of SIRT1 with resveratrol significantly reduces motor incoordination of MJD mice. The pharmacological SIRT1 activation could provide important benefits to treat MJD patients.

## Abstract (web summary)

SIRTs have been reported to provide neuroprotective actions in polyglutamine diseases, and are linked to the beneficial effects of caloric restrictive diets. Here, the authors show caloric restriction improves behavioural and neuropathological deficits in MJD model mice, an effect dependent on SIRT1 activity.

## Graphical abstract

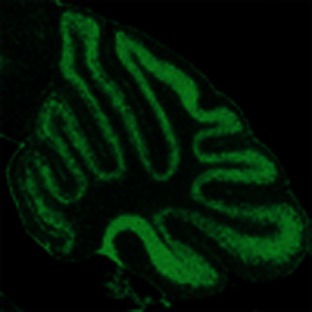

The polyglutamine (polyQ) diseases are a group of genetic neurodegenerative pathologies triggered by an over-repetition of the trinucleotide cytosine–adenine–guanine within the open reading frame of different genes and encoding long polyQ tracts in the respective proteins. The translated proteins tend to misfold and aggregate causing dysfunction and degeneration of specific neuronal subpopulations.

Machado–Joseph disease (MJD), also known as spinocerebellar ataxia type 3 (ref. 1) is one of these polyglutamine diseases, whose mutation is mapped to chromosome 14q32.1 (ref. 2). Despite rare, it is the most common autosomal dominantly inherited ataxia and reaches high prevalence in certain regions of Portugal, in particular in the islands of Azores^3^,^4^. The disorder has its onset at adult age and involves neurodegeneration within cerebellar systems, *substantia nigra*, cranial nerve motor nuclei^5^,^6^ and the striatum^7^,^8^, resulting in clinical hallmarks that include motor incoordination, postural instability, dysarthria and dysphagia among other symptoms^9^. Neurodegeneration is associated with accumulation of the polyglutamine-expanded ataxin-3 (stretch over 55 repeats)^10^ in the cells^11^,^12^. To this contributes the cleavage of the mutated protein in toxic fragments, mediated by calpains^13^,^14^,^15^ and the inefficient activation of autophagy to clear the mutant ataxin-3 (refs 16, 17). There is no treatment to prevent or slow the progression of MJD and only symptomatic treatments for the disease currently exist.

Caloric restriction (CR) is a dietary regimen involving consumption of fewer calories than usual but containing all the essential nutrients, without malnutrition. Since 1935 it is known that CR increases longevity in rats^18^ and over the last years it has been proved that CR extends lifespan in a wide spectrum of species^19^. Emerging data show that this effect is conserved in non-human primates, although some controversy subsists^20^,^21^,^22^.

The role of CR in neuroprotection was first revealed in 1990, when Park *et al*.^23^ showed that CR attenuated the age-related loss of spinal ganglion neurons in mice^23^ and its application in neurodegenerative disorders has been explored from that time on^24^,^25^,^26^. How CR exerts its beneficial effects has been the object of several hypotheses and its knowledge may contribute to open new therapeutic strategies for neurodegenerative diseases and other age-related diseases.

Sir2 or silent information regulator 2 was found in yeast and its mammalian orthologues are known as sirtuins (SIRTs). SIRTs are NAD^+^-dependent deacetylases found to slow ageing in yeast and higher organisms, including mammals^27^,^28^,^29^. Their activity is required for effects of CR in many organisms^30^. In mammals, seven SIRTs have been identified, namely SIRT1–7. Each SIRT has a specific cellular localization and function. SIRT1 is the most closed orthologue of *Sir2*, as *Sir2* is a NAD^+^-dependent deacetylase that is mainly located in the nucleus^31^ and is so far the most studied member in this family. Moreover, SIRT1 is activated by some compounds, such as, resveratrol^32^. It has been reported that SIRT1 plays a neuroprotective role in Huntington's disease^33^,^34^, and spinal and bulbar muscular atrophy^35^, both polyglutamine diseases. Nevertheless, until now the protective role of SIRT1 in other polyglutamine diseases, namely spinocerebellar ataxias, had not been investigated.

In the present study we provide compelling evidence that CR not only robustly mitigates the neuropathology but also rescues motor impairments in mouse models of MJD. The knowledge of the main mechanism(s) underlying these effects is crucial to develop a new therapeutic strategy to be applied in humans. We show that CR alleviation of MJD is mediated by SIRT1, highlighting the benefits of increasing SIRT1 expression or activity to alleviate this and potentially other spinocerebellar ataxias.

## Results

### CR drastically alleviates motor deficits in MJD mice

Previously, Torashima *et al*.^36^ showed that the transgenic (Tg) MJD mouse model used in this study exhibits a remarkable cerebellar atrophy and a severe loss of motor coordination and balance, since at least the third week of age^36^. In the present study, the motor coordination and balance of Tg MJD mice maintained with *ad libitum* (AL) diet or with CR diet were evaluated by beam walking test, stationary and accelerated rotarod tests, pole test and swimming test. We started the CR regimen in 6-week-old mice, thus in a postsymptomatic stage of the disease. To follow the effects of CR during the progressive stages of the disease, behavioural outcomes were measured every 3 weeks until the end of the study.

**Figure 1:**
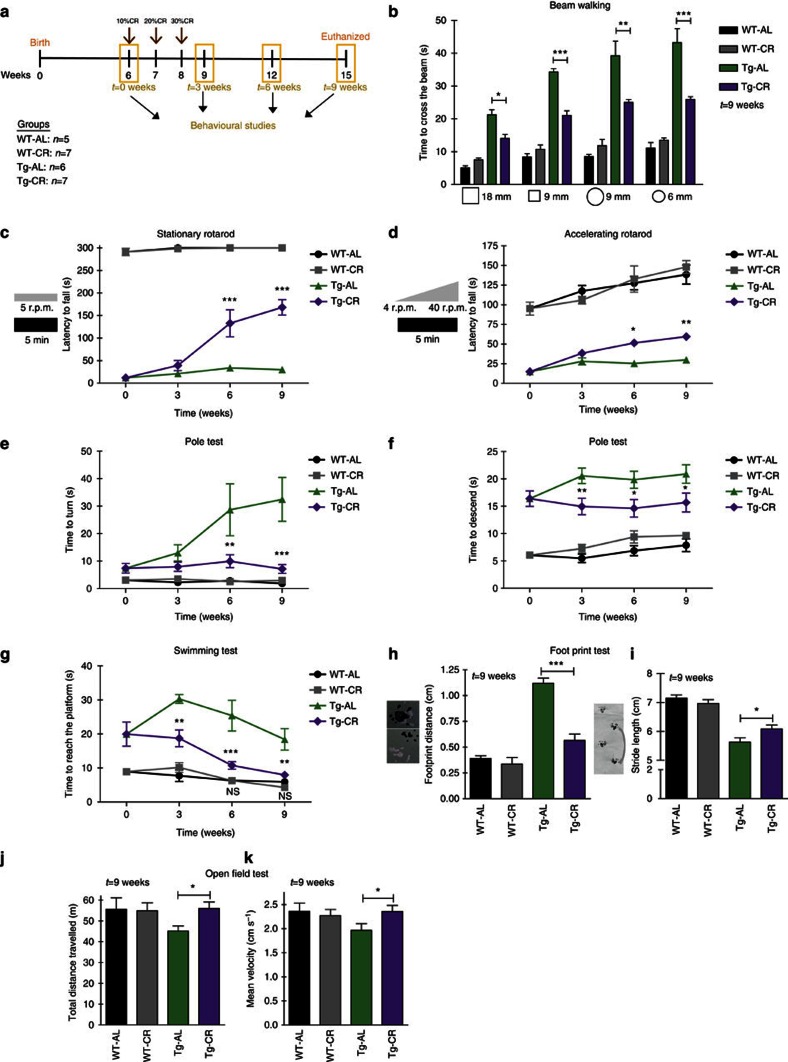
CR alleviates balance and motor coordination impairments and ameliorates gait and locomotor activity in a Tg MJD mouse model. (**a**) Representative study design; 6-week-old Tg and WT littermate mice were divided in two groups: AL group (AL) and calorie-restricted group (CR). Every 3 weeks, behavioural tests were performed, until animals were 15 weeks old. (**b**) Beam walking test demonstrating an amelioration of the motor performance of calorie-restricted mice, 9 weeks after the beginning of CR diet. (**c**,**d**) Tg MJD mice in CR diet had better performance in constant velocity (5 r.p.m.) and accelerated (from 4 to 40 r.p.m.) rotarod, compared with Tg MJD AL mice. (**e**,**f**) Vertical pole test. Since the sixth week, calorie-restricted Tg MJD mice turned downwards easier than the Tg mice in the AL diet (**e**) and descended the beam with less difficulties (**f**). (**g**) Swimming test performance. Since the sixth week, Tg mice exhibited a rescue of the common improper performance in the swimming pool. (**h**,**i**) Quantitative analysis of the footprint patterns. Calorie-restricted mice had a higher stride length and a more perfect footprint overlap and their performance is significantly closer to WT performance, 9 weeks after the beginning of CR diet. (**j**,**k**) Open-field test. Calorie-restricted Tg MJD mice travelled the same distance and with the same mean velocity than WT mice. Calorie-restricted mice had a higher stride length and a more perfect footprint overlap, and their performance is significantly closer to WT performance, 9 weeks after the beginning of CR diet. Data represent mean±s.e.m.; NS *P*>0.05, *P<0.05, **P<0.01 and ***P<0.001 compared with Tg-AL. (**b**–**g**) Two-way analysis of variance (ANOVA) with Bonferroni's post-hoc test. (**h**–**k**) One-way ANOVA with Bonferroni's post-hoc test. WT-AL *n*=5; WT-CR *n*=7; Tg-AL *n*=6; Tg-CR *n*=7. See also Supplementary Fig. 1.

Six weeks after the beginning of CR diet, Tg MJD mice showed a clear improvement in motor coordination and balance, evaluated by beam walking test, which reached significance for the 6 and 9 mm, round and square beams at this time point (Supplementary Fig. 1). At 9 weeks' time point, 15-week-old, calorie-restricted Tg MJD mice showed a robust improvement of motor coordination and balance, compared with AL mice (Fig. 1b).

To further assess motor coordination and balance, stationary and accelerated rotarod tests were performed. In stationary rotarod, Tg mice maintained in the AL diet quickly fell off the stationary rotarod, whereas calorie-restricted Tg mice exhibited an impressive improvement of performance (4.5 times) from the sixth week onwards (Fig. 1c; *t*=6 weeks; Tg-AL: 33.7±7.0 s versus Tg-CR: 132.7±29.9 s). Similar results were obtained with the accelerated rotarod (Fig. 1d; Tg-AL: 25.3±3.0 s versus Tg-CR: 51.4±4.2 s), which persisted along the experimental time course in both protocols.

This improvement in motor coordination and balance was additionally investigated with the pole test. Tg MJD mice in the control group showed a remarkably prolonged *t*-turn, compared with the calorie-restricted group, from the sixth week onwards (Fig. 1e; *t*=6 weeks; Tg-AL: 28.7±9.4 s versus Tg-CR: 10.0±2.4 s). Moreover, calorie-restricted Tg mice descended the vertical beam faster than mice in the AL diet and this difference was statistically different since the third week (Fig. 1f; *t*=3 weeks; Tg-AL: 20.6±1.4 s versus Tg-CR: 15.0±1.5 s) and was maintained along the experimental time.

Using the swimming test, we observed that calorie-restricted MJD Tg mice, since the third week, swam faster and showed less difficulties to reach the platform, in comparison with the control group (Fig. 1g; *t*=3 weeks; Tg-AL: 30.3±1.3 s versus Tg-CR: 18.7±2.4 s). Importantly, at 6 and 9 weeks after the beginning of the study, performance of calorie-restricted Tg MJD mice did not differ from the performance of wild-type (WT) mice, suggesting that a complete rescue of the phenotype occurred (Fig. 1g; *t*=6 weeks; Tg-CR: 10.8±1.1 s versus (WT-CR: 6.3±0.8 s or versus *n*=5 WT-AL: 6.3±0.9 s)).

Furthermore, gait was studied by analysis of footprint patterns. Nine weeks after the beginning of the CR diet, Tg mice in the CR diet showed a rescue of the affected footprint overlap (Fig. 1h; *t*=9 weeks; Tg-AL: 1.12±0.05 cm versus Tg-CR: 0.57±0.06 cm; WT-AL: 0.39±0.028 cm versus Tg-CR: 0.57±0.06 cm; *P*>0.05). A similar outcome was registered on the analysis of stride length. Calorie-restricted Tg MJD mice showed a consistently longer stride length in comparison with the control group (Fig. 1i; Tg-AL: 5.6±0.15 cm versus Tg-CR: 6.09±0.14 cm). These results show a significant improvement in the affected gait of Tg MJD mice with CR diet.

Finally, horizontal locomotor activity was evaluated by the open-field test. Calorie-restricted Tg MJD mice completely reverted the typical hypoactivity of this model (Fig. 1j), travelling consistently longer distances than the AL mice (Fig. 1j; *t*=9 weeks; Tg-AL: 45.15±2.40 m versus Tg-CR: 56.02±3.11 m), with a higher mean velocity (Fig. 1k; *t*=9 weeks; Tg-AL: 1.972.0±0.14 cm s^−1^ versus Tg-CR: 2.36±0.12 cm s^−1^), in both cases reaching the values observed for WT mice. Altogether, these results show that CR improves locomotor activity.

Importantly, no differences were found for WT mice submitted to CR or AL diets in any behaviour test. This finding suggests that CR animals do not perform better in these tests simply because they are leaner. Altogether, these data demonstrate that 9 weeks of CR robustly reduces the severe motor deficits in a postsymptomatic Tg mouse model of MJD.

### CR ameliorates neuropathology of MJD mice

**Figure 2:**
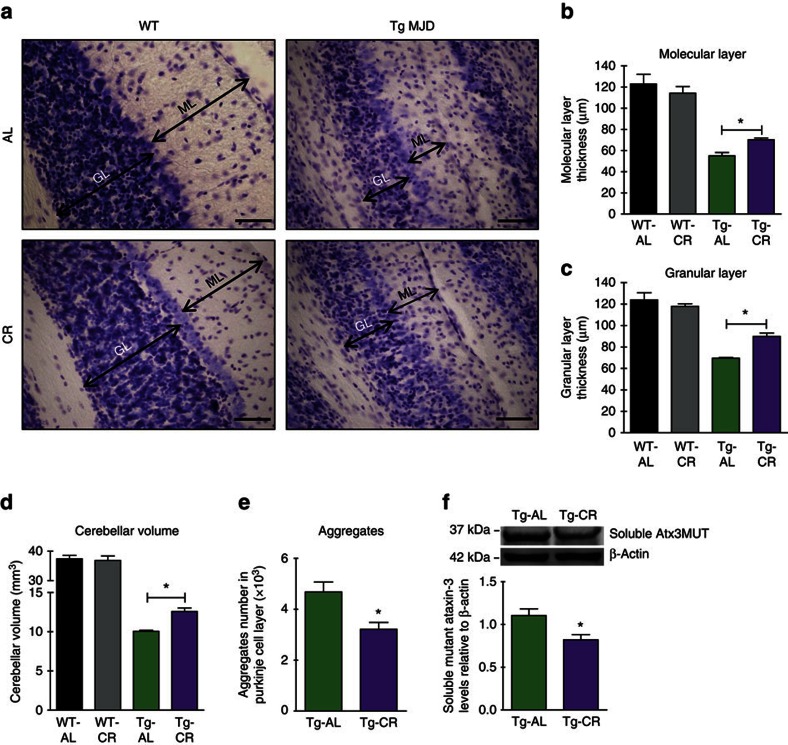
Nine weeks of CR prevents cerebellar neuropathology in Tg MJD mice. (**a**) Cresyl violet-stained sections demonstrate the thickness of molecular layer (ML) and granular layer (GL) of lobule V of cerebellum in 15-week-old littermate WT and Tg MJD mice in AL or CR diet, quantified in **b** and **c**. Scale bar, 50 μm. (**d**) Quantification of cerebellar volume showed a significantly higher cerebellar volume in calorie-restricted mice. (**e**) Analysis of the number of mutant ataxin-3 inclusions in the cerebellar Purkinje cells of Tg MJD mice. The total number of aggregates in Purkinje cells is significantly lower in calorie-restricted mice, in comparison with AL mice. (**f**) Relative levels of the soluble form of mutant ataxin-3 normalized with β-actin were significantly decreased in Tg mice in the CR diet. Data represent mean±s.e.m.; NS *P*>0.05 and *P<0.05 compared with Tg-AL. (**b**–**d**). One-way analysis of variance with Bonferroni's *post-hoc* test. (**e**,**f**) Unpaired Student's *t*-test. WT-AL *n*=3; WT-CR *n*=4; Tg-AL *n*=4; Tg-CR *n*=4.

Next, we wanted to investigate whether motor improvements, triggered by CR, correlate with histopathological changes in the cerebellum. Tg MJD mice used in this study present severe cerebellar atrophy and Purkinje cells are specifically affected^36^. Representative images of cerebellar lobule V confirm the generalized cerebellar atrophy in Tg MJD mice (Fig. 2a). We evaluated neuropathology at the last time point (9 weeks after the beginning of CR) when we observed the most prominent CR effects in motor behaviour.

In agreement with the behavioural data, 15 weeks old calorie-restricted Tg MJD mice exhibited significantly larger layers thickness in comparison with AL mice (Fig. 2b; molecular layer: Tg-AL: 55.1±3.0 μm and Tg-CR: 70.3±1.5 μm; Fig. 2c; granular layer: Tg-AL: 69.5±0.7 μm versus Tg-CR: 89.9±3.2 μm), which suggests prevention of neurodegeneration. In contrast, no difference was found in layer thickness within the two groups of WT mice (Fig. 2b; molecular layer: WT-AL: 122.9±3.1 μm; WT-CR: 114.3±6.2 μm; Fig. 2c; granular layer: WT-AL: 123.8±6.7 μm versus WT-CR: 117.9±2.5 μm). Cerebellar volume was also evaluated and calorie-restricted Tg MJD mice had a significantly larger cerebellar volume in comparison with the AL Tg group (Fig. 2d; Tg-AL: 10.1±0.1 mm^3^ versus Tg-CR: 12.6±0.4 mm^3^). Again, there was no difference between the two groups of WT mice (Fig. 2d; *n*=3 WT-AL: 37.4±1.2 mm^3^ versus *n*=4 WT-CR: 36.9±1.5 mm^3^).

The polyQ expansion in mutant ataxin-3 leads to the formation of intranuclear inclusions, one of the main hallmarks of MJD^12^,^37^. Therefore, we investigated whether beneficial effects of CR in motor abnormalities are related with changes in the number of mutant ataxin-3 aggregates in the cerebellum. The number of aggregates in Purkinje cells of the cerebellum in calorie-restricted Tg mice was reduced around 30% in comparison with the AL Tg mice (Fig. 2e; Tg-AL: 4680±393 versus Tg-CR: 3214±271) and the levels of soluble mutant ataxin (Fig. 2f).

Altogether, these results demonstrate that CR prevents cerebellar neurodegeneration in MJD mice, while it simultaneously decreases the levels of mutant ataxin-3.

### CR reverts abnormal decrease of SIRT1 levels

As increases in SIRT1 levels and activity are intrinsically related to the effects on lifespan and neuroprotection mediated by CR^38^,^39^, we evaluated the messenger RNA and protein levels of SIRT1 in the cerebellum of 15-week-old WT versus AL or calorie-restricted MJD Tg mice.

**Figure 3:**
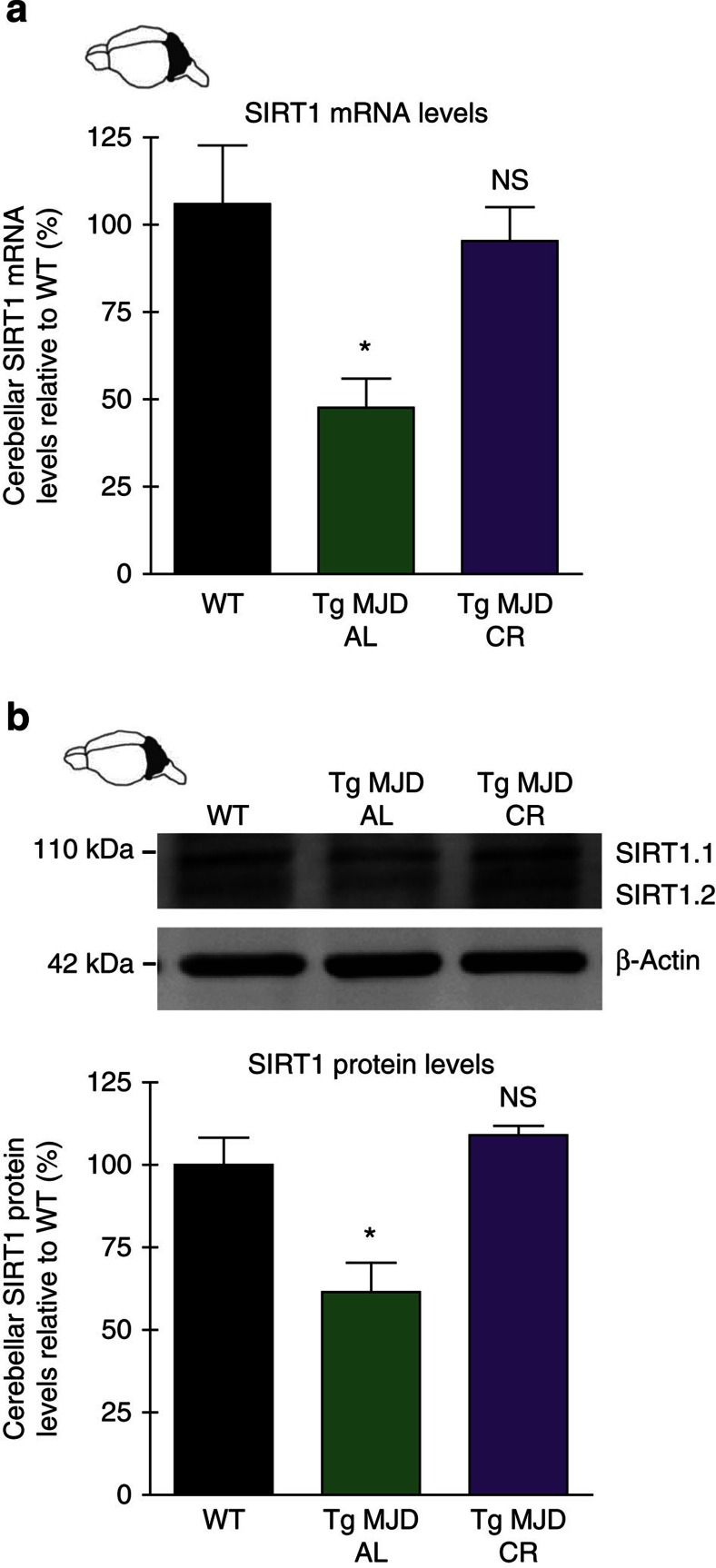
SIRT1 levels are abnormally reduced in the cerebellum of MJD Tg mouse model and CR re-establishes normal SIRT1 cerebellar levels. (**a**) SIRT1 is compromised in the cerebellum of Tg MJD mice. A significant decrease of 58.4±8.3% in mRNA levels of SIRT1 was detected. These results were supported by western blotting in (**b**). Data represent mean±s.e.m.; NS *P*>0.05 and *P<0.05 compared with Tg-AL. (**a**,**b**) WT *n*=5; Tg-AL *n*=5; Tg-CR *n*=4. SIRT1.1 (SIRT1 isoform 1) and SIRT1.2 (SIRT1 isoform 2). See also Supplementary Fig. 2.

A dramatic decrease of 58.4±8.3% in mRNA levels and of 38.5±8.8% in protein levels of SIRT1 in Tg MJD mice, in comparison with WT littermate (Fig. 3a,b), was observed. The same results were observed in human fibroblasts of MJD patients in comparison with healthy patients (data not shown). On CR, SIRT1 mRNA and protein levels were completely re-established to the levels observed in the non-diseased mice (Fig. 3a,b). As expected, CR also induced the activation of SIRT1, evaluated by the decrease in acetylated levels of the SIRT1 substrate, Forkhead box protein O1 (Supplementary Fig. 2).

Overall, these results suggest that SIRT1 expression is compromised in the cerebellum of Tg MJD mice and CR can revert this defect, reinstating normal SIRT1 levels.

### SIRT1 overexpression ameliorates MJD neuropathology

**Figure 4:**
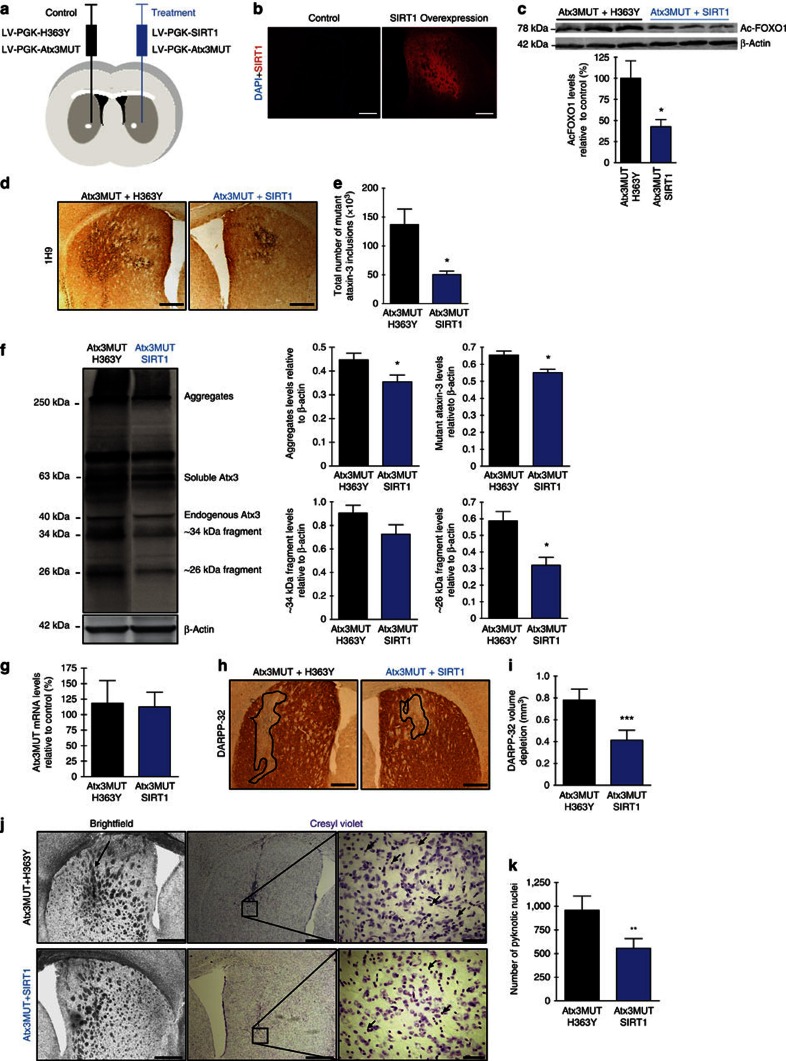
SIRT1 overexpression reduces mutant ataxin-3 toxicity and neuronal dysfunction in a striatal lentiviral model of MJD. (**a**) Schematic representation of the strategy used to create *in vivo* striatal lentiviral model of MJD and to overexpress SIRT1/H363Y. Five-week-old C57Bl/6J mice were bilaterally injected with lentiviral vectors encoding for mutant ataxin-3. In the left hemisphere were co-injected lentiviral vectors encoding for H363Y and in the right hemisphere for SIRT1. Four weeks after the surgery, mice were euthanized. (**b**) SIRT1 immunoreactivity in the striatum of animals of the striatal lentiviral model of MJD co-injected with H363Y or SIRT1 versus a control animal. DAPI, blue, nuclei. Scale bar, 500 μm. (**c**) Acetylated Forkhead box protein O1 (FOXO1) levels are significantly lower in the striata of lentiviral model of MJD when the active form of SIRT1 was overexpressed, in comparison with control side where the inactive form of SIRT1 was overexpressed. (**d**) With an anti-ataxin-3 antibody (Ab 1H9), total number of mutant ataxin-3 inclusions were counted and are quantified in **e**. Scale bar, 500 μm. (**f**) Western blot analysis demonstrated that SIRT1 overexpression significantly reduced several toxic forms of mutant ataxin-3, namely aggregates, soluble form and ∼34 and ∼26 kDa fragments. (**g**) Quantitative reverse transcriptase–PCR analysis of Atx3MUT mRNA. (**h**) DARPP-32 staining revealed a major loss of DARPP-32 immunoreactivity in the striatal hemisphere co-infected with mutant ataxin-3 and H363Y. Scale bar, 500 μm. This was quantified in **i** as depleted volume of DARPP-32 staining. (**j**) Cresyl violet staining indicating pyknotic nuclei in both hemispheres. A higher number of pyknotic nuclei were visible around the injection site area in the H363Y-transduced hemisphere. Scale bar, 500 μm (brightfield and left cresyl violet) and 50 μm (right cresyl violet). This was quantified in **k**. Data represent mean±s.e.m. *P<0.05, **P<0.01 and ***P<0.001 compared with non-treated hemisphere. (**c**,**e**–**g**,**i**,**k**) Paired Student's *t*-test. *n*=4.

Previously, we showed that CR robustly mitigated MJD phenotype and reverted the compromised cerebellar levels of SIRT1 in mice. We then investigated whether lentiviral overexpression of SIRT1 would be sufficient to mimic the effects of CR and mediate similar alleviation of MJD pathology. For this purpose, we took advantage of a genetic mouse model of MJD generated by localized lentiviral-mediated expression of mutant ataxin-3 in the striatum, another affected brain region in MJD^7^,^13^. This model is particularly useful, as it allows a precise quantification of neuropathology regarding loss of the Dopamine- and cyclic AMP-regulated phosphoprotein 32 (DARPP-32) marker and number of ataxin-3 aggregates in specific regions of interest. Therefore, in 5-week-old WT mice we stereotaxically co-injected in the left hemisphere lentiviral vectors encoding for mutant ataxin-3 and an inactive SIRT1 mutant—H363Y—as control (control hemisphere), whereas in the right hemisphere we co-injected lentiviral vectors encoding for mutant ataxin 3 and for SIRT1 (Fig. 4a). SIRT1 overexpression was specifically located at the striatum and, as expected, induced a decrease in the deacetylation levels of SIRT1 substrate—Forkhead box protein O1—in the striatum (Fig. 4b,c).

Insoluble aggregates of ataxin-3, despite the unclear role in the pathology, are a hallmark of the disease that can be used as a surrogate marker. By immunohistochemistry (Fig. 4d,e) and by western blotting (Fig. 4f), we observed that SIRT1 overexpression induced a 60.9±4.8% decrease in the total number of aggregates (Fig. 4e; non-treated: 136,960±26,808 versus SIRT1 50,480±5,960) and a significant decrease in the aggregated and soluble forms of mutant ataxin-3 (Fig. 4f). Interestingly, the ∼34 and ∼26 kDa toxic fragments, resulting from processing of mutant ataxin-3 (ref. 13), were also decreased in the hemisphere where SIRT1 was overexpressed, indicating an interference of SIRT1 in the process of formation or degradation of these fragments. No changes were observed regarding the mRNA levels of mutant ataxin-3 (Fig. 4g). Altogether, these results suggest that SIRT1 overexpression decreases accumulation and aggregation of mutant ataxin-3 in intranuclear inclusions and decreases the levels of toxic fragments, contributing to the decrease of mutant ataxin-3 toxicity.

In the control hemisphere DARPP-32 immunoreactivity decreased with a depleted staining volume of 0.78±0.10 mm^3^, whereas in the contralateral hemisphere SIRT1 overexpression reduced the volume depletion of DARPP-32 by 52% (0.41±0.09 mm^3^; Fig. 4h,i), clearly demonstrating its neuroprotective activity.

Furthermore, bright-field images showed a small condensation of the internal capsule of the striatum, triggered by neuronal dysfunction induced by mutant ataxin-3, in the control hemisphere, which was not observed in the hemisphere where SIRT1 was overexpressed (Fig. 4j, left). Importantly, SIRT1 overexpression reduced by 42.0±10.7% the number of pyknotic nuclei (Fig. 4k). These data suggest that SIRT1 prevents cell injury and striatal degeneration triggered by mutant ataxin-3.

### SIRT1 overexpression decreases MJD neuroinflammation

To explore the mechanism(s) by which SIRT1 overexpression produced beneficial effects on MJD mouse models, we investigated whether SIRT1 interferes with the characteristic neuroinflammation observed in the lentiviral MJD mouse model^7^,^40^.

**Figure 5:**
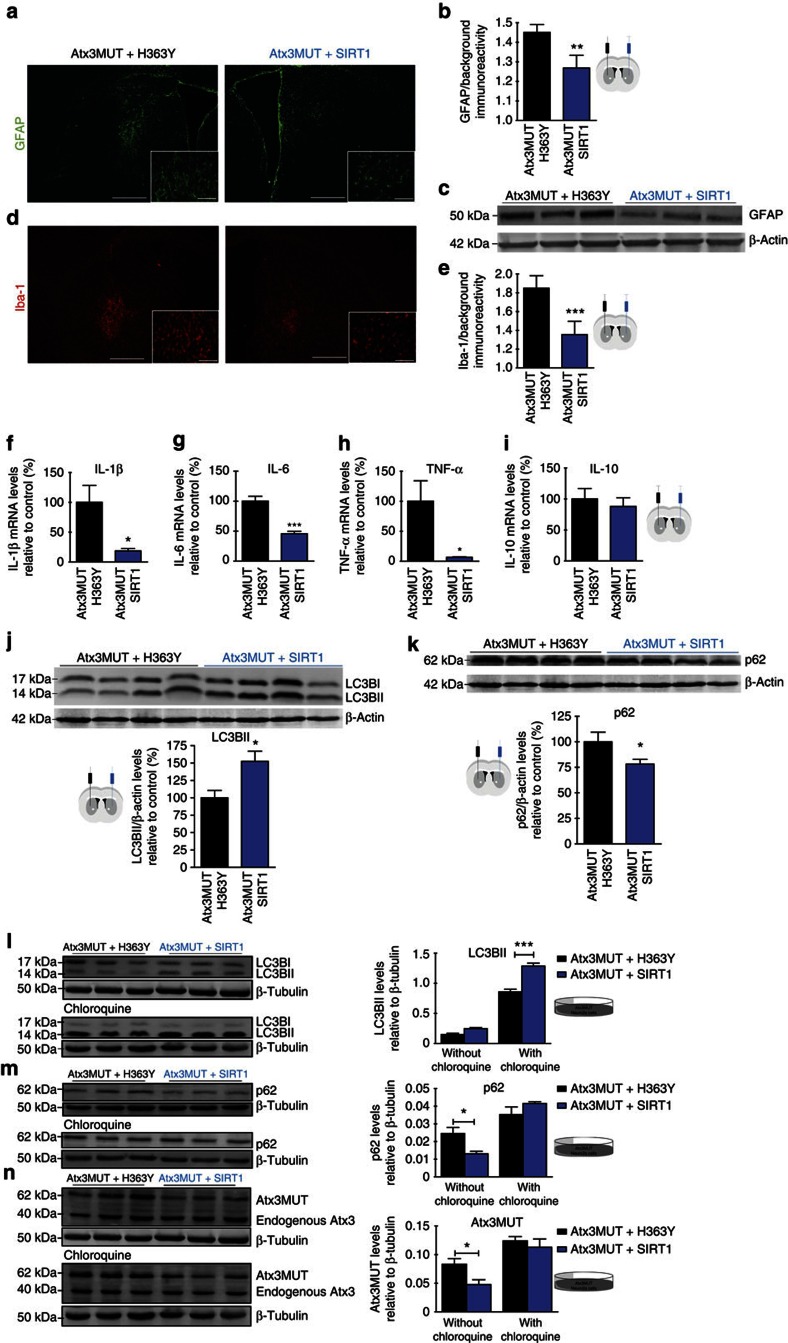
SIRT1 overexpression reduces MJD-associated neuroinflammation and activates autophagy. (**a**–**e**) Quantitative analysis of glial fibrillary acidic protein (GFAP) and ionized calcium binding adaptor molecule 1 (Iba-1) immunoreactivity at 4 weeks after the co-injection of the viral vectors in the striatal lentiviral model of MJD. (**a**–**c**) SIRT1 decreased astrocytic activation induced by mutant ataxin-3, evidenced by the decrease in GFAP immunoreactivity and by the reduction in GFAP protein levels, evaluated by western blotting in **c**. (**d**,**e**) SIRT1 reduced microglia recruitment triggered by mutant ataxin-3, supported by the decrease of Iba-1 immunoreactivity, in comparison with the control hemisphere. Scale bar, 500 μm in larger images and 100 μm in smaller images. (**f**–**i**) mRNA levels of inflammatory cytokines were evaluated by quantitative reverse transcriptase–PCR. (**f**–**h**) mRNA levels of pro-inflammatory IL-1β, IL-6 and TNF-α cytokines were significantly decreased in the hemisphere where SIRT1 was overexpressed. (**i**) mRNA levels of anti-inflammatory IL-10 cytokine were not changed with SIRT1 overexpression. (**j**,**k**) SIRT1 overexpression activates autophagic flux in the striatum of striatal lentiviral model of MJD. (**j**) SIRT1 overexpression induces an increase in LC3BII levels, in comparison with the overexpression of H363Y. (**k**) SIRT1 overexpression reduces significantly p62 levels, in comparison with the hemisphere where H363Y was overexpressed. (**l**–**n**) Neuro 2a cells were co-infected with lentivirus encoding for mutant ataxin-3 and for H363Y (control condition) or co-infected with lentivirus encoding for mutant ataxin-3 and for SIRT1. (**l**) SIRT1 overexpression in the presence or absence of an autophagic flux inhibitor increased LC3BII levels. (**m**) In the absence of chloroquine, p62 levels were increased and did not change in the presence of the lysosomal inhibitor. (**n**) Mutant ataxin-3 levels were reduced when SIRT1 was overexpressed in the cells, in the absence of chloroquine. In the presence of chloroquine, the changes were not observed. Data represent mean±s.e.m. *P<0.05 , **P<0.01 and ***P<0.001 compared with control. (**b**,**e**–**k**). Paired Student's *t*-test. *n*=4. (**l**–**n**) Two-way analysis of variance with Bonferroni's *post-hoc* test. *n*=4. See also Supplementary Figs 3–5.

In non-treated hemispheres, we observed a local glial fibrillary acidic protein immunoreactivity increase, suggestive of astrocytic activation. SIRT1 overexpression reduced glial fibrillary acidic protein immunoreactivity (Fig. 5a–c). In addition, in the non-treated hemisphere, a strong immunoreactivity for the microglial marker Iba-1 was observed, revealing microglial recruitment, which was robustly and significantly reduced in the hemisphere where we overexpressed SIRT1 (Fig. 5d,e). These results suggest that SIRT1 overexpression prevents reactive gliosis associated with MJD.

Furthermore, we analysed the changes in the mRNA levels of inflammatory cytokines, namely interleukin (IL)-1β, IL-6, tumour necrosis factor-α (TNF-α) and IL-10. We found a strong and significant decrease in the mRNA levels of pro-inflammatory cytokines (IL-1β, IL-6 and TNF-α) in the hemisphere, where we overexpressed SIRT1 relative to the non-treated hemisphere (Fig. 5f–h). Interestingly, the levels of one anti-inflammatory cytokine, IL-10, were not different between non-treated and SIRT1-overexpressing striata (Fig. 5i). These results suggest that SIRT1 has a specific downregulating effect over pro-inflammatory cytokines, but does not have effects on the anti-inflammatory network.

Overall data indicate that, at least, one of the mechanisms by which SIRT1 ameliorates neuropathology of MJD in striatal lentiviral model of MJD is by the downregulation of neuroinflammatory markers.

### *In vitro* and *in vivo* SIRT1 overexpression activates autophagy

SIRT1 deacetylase activity is important for autophagy activation induced by starved conditions^41^. In addition, we previously demonstrated that autophagy is compromised in MJD and its activation can alleviate MJD^16^,^17^. Therefore, we hypothesized that SIRT1 increases mutant ataxin-3 clearance by activating autophagy.

First, to explore whether autophagy could be activated by CR, we evaluated LC3BII and p62 protein levels in the cerebella of 15-week-old Tg animals fed during 9 weeks with a CR versus AL diet. We observed a significant increase in LC3BII levels and a decrease in p62 levels in cerebella of MJD Tg mice fed with CR diet, in comparison with MJD TgAL- fed animals (Supplementary Fig. 3). Moreover, we evaluated the same parameters in the striatum of the lentiviral model of MJD mice where SIRT1 was overexpressed in comparison with the overexpression of a non-functional SIRT1 (H363Y). Interestingly, we also observed a significant increase in LC3BII and a decrease in p62 levels, in the striatum where SIRT1 was overexpressed in comparison with H363Y overexpression. These results suggest that in fact SIRT1 activates autophagy in MJD mice (Fig. 5j,k). To further assess more precisely what SIRT1 does to autophagic flux, we performed an *in vitro* study, using Neuro2a cells expressing mutant ataxin-3 in the presence and absence of a lysosomal inhibitor—chloroquine. Interestingly, in cells overexpressing SIRT1, we observed an increase in LC3BII levels, in comparison with control cells (Fig. 5l). To confirm whether SIRT1 is activating autophagy, we evaluated LC3BII levels in the presence of the lysosomal degradation inhibitor (chloroquine 100 μM) and again an increase in LC3BII levels was observed, as compared with control condition (Fig. 5l). Cells overexpressing SIRT1 also exhibited a significant decrease in p62 or sequestosome 1 (SQSTM1), in comparison with control condition (Fig. 5m), which, as expected, was prevented in the presence of chloroquine. In addition, SIRT1 overexpression induced a decrease of mutant ataxin-3, which was prevented by chloroquine (Fig. 5n).

Furthermore, to better understand the direct effect of SIRT1 on autophagy pathway and on MJD pathology, we performed additional experiments using the same *in vitro* model of MJD that is suitable to address autophagy features—Neuro2a cells expressing mutant ataxin-3. We generated a short hairpin RNA (shRNA) targeting ATG5 in addition to the shRNA targeting SIRT1. In the Neuro2a cell line expressing a mutated form of ataxin-3, we genetically silenced SIRT1 or ATG5 in comparison with a control shRNA. We evaluated by western blotting, the protein levels of LC3BII and p62. As expected, we observed that genetic silencing of ATG5 significantly decreased the conversion of LC3BI to LC3BII (Supplementary Fig. 4a) and induced an accumulation of p62 (Supplementary Fig. 4b) and consequently mutant ataxin-3 (Supplementary Fig. 4c). Similar results were obtained on autophagy inhibition by SIRT1 knockdown, indicating that SIRT1 inhibition causes autophagic flux disruption. To further clarify the mechanism through which SIRT1 deacetylating activity stimulates autophagy and clears mutant ataxin-3, in cells expressing mutant ataxin-3, we genetically silenced ATG5 (or not) and overexpressed SIRT1 or the non-functional SIRT1—H363Y. Interestingly, we made the following observations: (1) as expected, when autophagy is not disrupted, SIRT1 activates autophagy, demonstrated by the increase in LCBII levels and the decrease in p62; (2) SIRT1 induces mutant ataxin-3 clearance and to do this its deacetylase activity is crucial, because its inactive form (H363Y) had no effects; (3) if autophagy is disrupted (here promoted by ATG5 genetic silencing), SIRT1 is not able to reduce mutant ataxin-3 levels, indicating that it is by activating autophagy that SIRT1 mediates the clearance of mutant ataxin-3 (Supplementary Fig. 5). This study indicates that in fact SIRT1 plays an important role in ataxin-3 clearance through the participation on autophagy, because the reduction of mutant ataxin-3 promoted by SIRT1 is reverted when ATG5 is genetically silenced.

Altogether, these results suggest that SIRT1 activates autophagy and this could result on mutant ataxin-3 clearance and consequently to the decrease in its levels.

### CR alleviates MJD by increasing SIRT1 levels

To investigate whether the mechanism of CR is mediated by SIRT1, we evaluated whether silencing SIRT1 would prevent the neuroprotective effects of CR in the striatal lentiviral model of MJD. Thus, we considered four conditions: (1) animals injected with vectors encoding mutant ataxin-3 and given AL diet, (2) the same but food restricted, (3) food restricted and co-injected in the left hemisphere with lentiviral vectors encoding for mutant ataxin-3 and a control shRNA and (4) food restricted and co-injected in the right hemisphere with lentiviral vectors encoding for mutant ataxin-3 and for a shRNA targeting mouse SIRT1.

**Figure 6:**
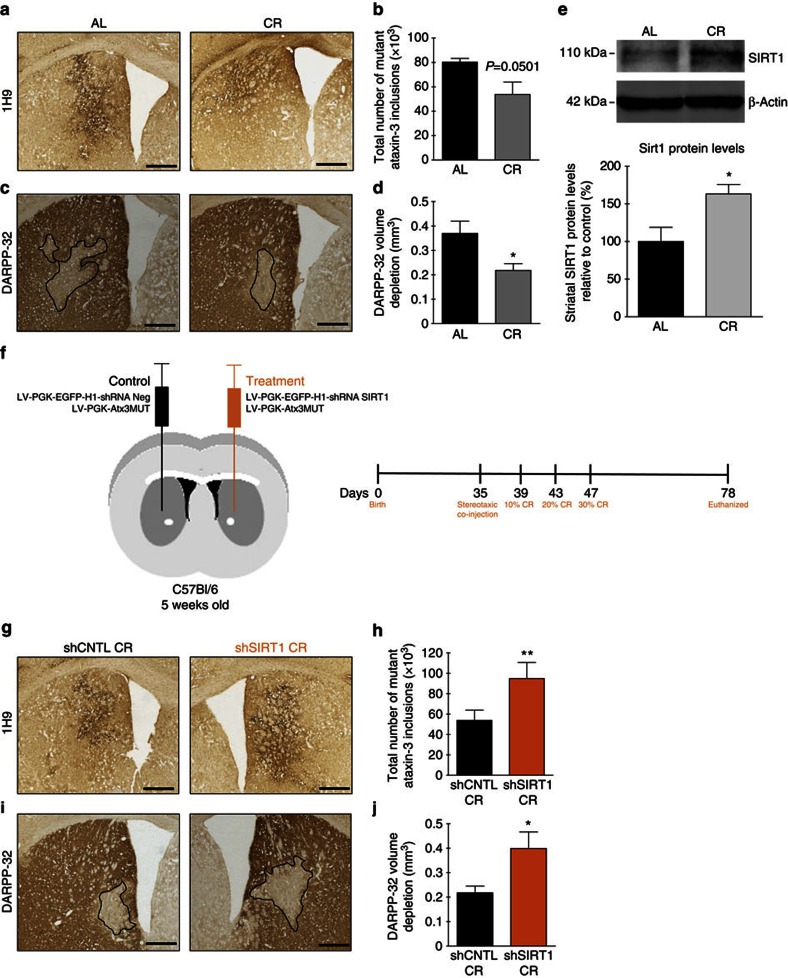
CR alleviates MJD neuropathology by the SIRT1 pathway in striatal lentiviral model of MJD. (**a**) Mutant ataxin-3 aggregates counting, quantified in **b**, showed that CR decreased mutant ataxin-3 inclusions in the striatum. Scale bar, 500 μm. (**c**) A higher DARPP-32 volume depletion, quantified in **d**, was observed in animals in an AL diet, compared with calorie-restricted mice. Scale bar, 500 μm. (**e**) Immunoblotting demonstrating that calorie-restricted mice have an increase in the protein levels of SIRT1 in striatum in comparison with AL mice. (**f**) Schematic representation of the study. Five-week-old C57Bl/6J mice were stereotaxically injected in the striatum. In each hemisphere we injected lentiviral vectors encoding for mutant ataxin-3; in the left hemisphere was co-injected lentiviral vectors encoding for the shRNA control and in the right hemisphere was co-injected lentiviral vectors encoding for the shRNA targeting SIRT1. Four days after the surgeries, animals were divided in two groups. One group of mice was maintained in an AL diet and the other was in a CR diet. (**g**) Mutant ataxin-3 inclusions counting showed that SIRT1 silencing prevented the decrease in the number of inclusions induced by CR. This was quantified in **h**. Scale bar, 500 μm. (**i**) DARPP-32 staining revealed that genetic silencing of SIRT1 in the striatal lentiviral model of MJD induced a blockage of the benefits of CR. This was quantified in **j**. Scale bar, 500 μm. Data represent mean±s.e.m.; *P<0.05 and **P<0.01 compared with control. (**b**,**d**,**e**) Unpaired Student's *t*-test. *n*=5. (**h**,**j**) Paired Student's *t*-test. *n*=5.

As expected, DARPP-32 volume depletion and the number of mutant ataxin-3 inclusions were significantly reduced in calorie-restricted mice compared with AL mice (Fig. 6a,b; AL: 80,200±3,040 versus 53,720±10,190; *P*=0.050; Fig. 6c,d; AL: 0.37±0.05 mm^3^ versus CR: 0.22± 0.03 mm^3^). Protein levels of SIRT1 were also increased by 63.2±12.5% in the striatum of calorie-restricted mice (Fig. 6e).

In contrast, silencing striatal SIRT1 (shSIRT1 CR) blocked the beneficial effects of CR as compared with control (shCNTL CR) (Fig. 6f–j). In fact, in SIRT1-silenced mice CR did not change the pathological striatal features induced by mutant ataxin-3, such as the number of aggregates in the striatum (Fig. 6b,h; AL: 80,200±3,040 versus shRNA SIRT1 CR: 94,830±15,810; *P*>0.05) neither DARPP-32 volume depletion (Fig. 6d,j; AL: 0.37±0.05 mm^3^ versus shRNA SIRT1 CR: 0.34±0.07 mm^3^; *P*>0.05), completely reverting the neuropathology to the one observed in AL mice.

Altogether, these results demonstrate that SIRT1 mediates the robust neuroprotective effects of CR in MJD.

### Resveratrol treatment improves motor behaviour in MJD mice

**Figure 7:**
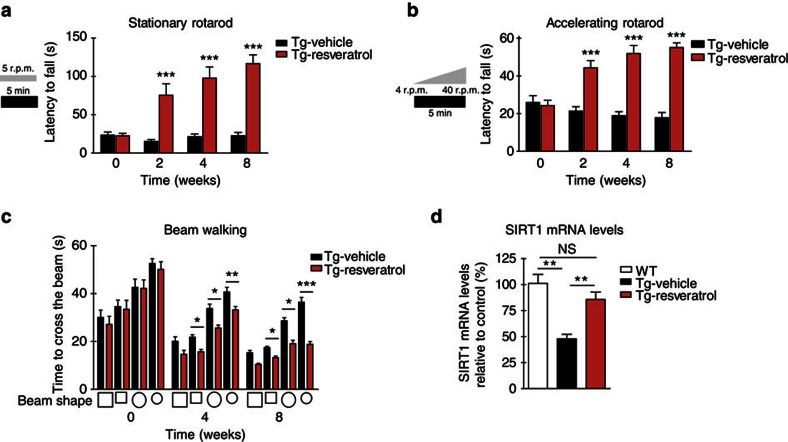
Peripheral administration of resveratrol ameliorates motor deficits and restores SIRT1 mRNA levels in Tg MJD mice. (**a**,**b**) Resveratrol-treated Tg MJD mice showed a significant better performance in constant velocity (5 r.p.m.) and accelerated (from 4 to 40 r.p.m.) rotarods, since the second week of treatment (eighth week after birth), compared with vehicle-treated Tg MJD mice. (**c**) Beam walking test with two square beams (18 or 9 mm width) and two round beams (9 or 6 mm diameter), demonstrating an amelioration of the motor performance promoted by resveratrol. (**d**) SIRT1 mRNA levels are decreased in Tg MJD animals (Tg-Vehicle) in comparison with WT mice (WT). SIRT1 mRNA levels are re-established with 8 weeks of daily treatment with 10 mg kg^−1^ body weight of resveratrol (Tg-Resveratrol). Data represent mean±s.e.m. (**a**–**c**) NS, nonsignificant; *P<0.05, **P<0.01 and ***P<0.001 compared with Tg vehicle-treated mice. Two-way analysis of variance (ANOVA) with Bonferroni's *post-hoc* test. Unpaired Student's *t*-test. *n*=8–9. (**d**) Data represent mean±s.e.m. NS. *P*>0.05 and **P<0.01. Relative to WT mice. One-way ANOVA with Bonferroni's *post-hoc* test. WT: *n*=6; Tg-Vehicle: *n*=5; Tg-Resveratrol: *n*=5.

In addition, we explored whether a pharmacological activator of SIRT1—the resveratrol—could be an effective therapy for the disease. We started the treatment with resveratrol in 6-week-old Tg mice, in a postsymptomatic stage of the disease. The results show that resveratrol decreases the motor deficits and imbalance of MJD mice observed in stationary and accelerated rotarod tests (Fig. 7a,b), and also in beam-walking test (Fig. 7c). Moreover, in Tg MJD mice we observed that resveratrol treatment was able to re-establish SIRT1 mRNA levels (Fig. 7d), in a similar way as CR did (Fig. 3a).

Altogether, these results suggest that resveratrol could be a potential pharmacological therapy to treat MJD patients.

## Discussion

In the present study we provide strong evidence demonstrating that CR robustly alleviates the neuropathology in two MJD mouse models (Tg and lentiviral MJD models) and rescues the motor impairments by a mechanism involving the upregulation of SIRT1, and consequent inhibition of neuroinflammation and activation of autophagy.

The cerebellum is one of the brain regions where neuropathology is most prominent in MJD and cerebellar damages cause motor incoordination, explaining the disabling clinical features of MJD^5^. The MJD Tg mice that we used in this study^36^ display a specific expression of the mutant ataxin-3 in Purkinje cells of the cerebellar cortex. Mice of this MJD model exhibit cerebellar atrophy and an early pronounced ataxic motor behaviour. We started the study after the development of the ataxic behaviour—in other words, in a postsymptomatic stage of the disease. In the present work, we show, with several behavioural tests, that CR strongly ameliorates the severe motor deficits of this Tg mouse model of MJD. Furthermore, in comparison with the AL-fed MJD mice, we observed that CR also decreases the neuropathology features of these mice, namely mediating an amelioration of cerebellar atrophy, molecular and granular layer thickness in the cerebellar cortex, while promoting a significant decrease in the aggregated and soluble forms of mutant ataxin-3 in Purkinje cells and in the cerebellar tissue. We suggest that the Purkinje cell preservation observed in calorie-restricted MJD mice resulted from the decrease in mutant ataxin-3 toxicity and this may explain the preserved larger molecular and granular layers thickness in comparison with AL-fed animals. Moreover, we also evaluated the effects in the striatal lentiviral model of MJD. In this model of MJD, we stereotaxically inject lentiviral vectors encoding for a mutated form of ataxin-3 in the striatum of 5-week-old mice, inducing neuropathology from the second week on, including striatal mutant ataxin-3 inclusions, synaptic loss and astrogliosis^3^,^6^,^7^,^8^,^9^. In the CR and in the SIRT1 lentiviral overexpression experiments using this model of the disease, we started the treatments at an early stage of the disease, namely before the development of the aforementioned alterations, complementing the observations made with the previous mouse model of the disease. Therefore, using two complementary MJD mouse models, we observed that CR and SIRT1 overexpression ameliorate the neuropathology in two stages of the disease: postsymptomatic and at an initial stage.

Some previous studies described benefits of CR in neurodegenerative disorders such as Parkinson's, Alzheimer's and Huntington's diseases^24^,^25^,^26^. Specifically, Qin *et al*.^26^ demonstrated that in Alzheimer's disease, the neuroprotection mediated by CR can be reproduced by manipulating cellular SIRT1 expression/activity, highlighting the role of SIRT1 in the effects of CR^26^. Another more recent study showed that CR induced the expression of SIRT1 and pharmacological activation of SIRT1 replicated the effects of CR in a neurodegeneration mouse model^39^. The increase in SIRT1 seems to be neuroprotective and, thus, to ameliorate neuropathology in some diseases. Interestingly, in the Tg mouse model of MJD used in the present study we observed that SIRT1 protein levels were decreased in the cerebellum, and that CR restored its levels, ameliorating MJD neuropathology. Besides, we wanted to investigate the potential beneficial effects resulting from SIRT1 overexpression in the striatum of MJD mouse model, to clarify the importance of SIRT1 in this mouse model of the disease. The results show that SIRT1 overexpression significantly reduced the characteristic MJD neuropathology highlighting the role and importance of SIRT1 in the disease.

The mechanism underlying the neuroprotective effects promoted by CR is not fully understood. In fact, there is a myriad of mechanisms described in which CR participates. It is described that CR increases the levels of neurotrophic factors and of stress-resistance proteins, such as heat shock protein 70, induces changes in regulatory proteins, namely in FOXOs proteins, and that these changes contribute to neuroprotection^24^,^25^,^42^. To further explore whether SIRT1 is the major mediator of CR beneficial effects, we genetically silenced SIRT1 in the striatum of AL versus calorie-restricted mice of the lentiviral model of MJD. We found compelling evidence demonstrating that CR drives its benefits mainly through the effects of SIRT1, due to the absence of neuroprotection in mice where SIRT1 was genetically silenced.

In recent times, we described that neuroinflammation and reactive gliosis is a feature of MJD in the two genetic mouse models of MJD that we used in this study^40^,^43^. Moreover, it was previously suggested that neuroinflammation, at least in part, underlies the neuroprotective action of CR and/or SIRT1 (ref. 44). Taking this information into account, we evaluated neuroinflammatory markers in the affected brain region of MJD mouse models after CR regimen and we observed in the striatum of the lentiviral MJD mouse model a significant decrease of gliosis markers and mRNA levels of pro-inflammatory cytokines (IL-1β, IL-6 and TNF-α), but no change on anti-inflammatory cytokine levels (IL-10). As others showed that SIRT1 deacetylates and inactivates nuclear factor-κB, a transcription factor that controls the gene expression of pro-inflammatory cytokines genes such as *IL-1β*, *IL-6* and *TNF-α* (refs 45, 46), our results suggest an effect of SIRT1 on the pro-inflammatory network.

It was also described that SIRT1 can activate autophagy, through the deacetylation and activation of important autophagy-related proteins, namely Atg5, Atg7 and Atg8 (ref. 47). Moreover, it was recently shown that SIRT1 plays a crucial role in autophagy, contributing to nuclear LC3 deacetylation, driving its transportation for the cytoplasm and the initiation of autophagy under starvation conditions^41^. Accordingly, we observed an activation of autophagy, triggered by SIRT1, which was specifically mediated by its deacetylase activity, given the absence of effects in the control condition (H363Y) where deacetylase activity was not present. We also observed a decrease in mutant ataxin-3 levels, suggesting that the reduction of mutant ataxin-3 toxicity is associated to autophagy activation and promotion of mutant ataxin-3 clearance. Thus, SIRT1 can deacetylate important autophagic substrates, leading to autophagy activation, mutant ataxin-3 clearance and a reduction in toxic fragments, thereby ameliorating neurodegeneration. The reduction in fragments described before could also be linked to the activation of autophagy. In fact, the faster turnover of soluble mutant ataxin-3 can contribute to the decrease in mutant ataxin-3 fragment levels. These results are in accordance to the absence of changes in mutant ataxin-3 mRNA, which is suggestive of a decrease of the different forms of ataxin-3 independently of transcriptional alterations.

In conclusion, the present study shows that SIRT1 activation strongly improves motor deficits and neuropathology in mouse models of MJD by SIRT1-mediated effects. Thus, a therapy based on SIRT1 activation and increasing SIRT1 levels, as resveratrol, is a promising approach for MJD therapy.

## Methods

### Animals and treatments

C57Bl/6 mice were obtained from Charles River, Spain, and C57Bl/6-background Tg MJD mice and age-matched WT control mice were previously described^36^,^48^; a colony of these Tg mice was established at the Centre for Neuroscience and Cell Biology of the University of Coimbra. Mice were housed under conventional 12-h light–dark cycle in a temperature-controlled room with water provided AL and food restricted or AL, depending on the group of study. Mice were fed with a standard AIN-93G diet. Since the sixth week after birth, in a postsymptomatic stage of the disease, calorie-restricted mice were fed with an amount of food equal to 90% of that consumed by mice in AL-fed corresponding group in the first period (10% of CR), 80% in the second period (20% of CR) and 70% from third period until the end of the study (30% of CR), without malnutrition. In resveratrol study, 6-week-old animals were intraperitoneally injected with vehicle or 10 mg kg^−1^ of body weight of resveratrol in 25% dimethyl sulfoxide in saline solution in a final volume of 50–70 μl adjusted for the body weight, daily during 8 weeks. The experiments were carried out in accordance with the European Community directive (2010/63/EU) for the care and use of laboratory animals. The researchers received adequate training (FELASA certified course) and certification to perform the experiments from Portuguese authorities (Direção Geral de Veterinária).

### Lentiviral vectors production

Lentiviral vectors encoding human mutant ataxin 3 (LV-PGK-Atx-3 72Q), human SIRT1 (LV-PGK-SIRT1), H363Y (LV-PGK-H363Y), negative shRNA (LV-PGK-EGFP-H1-shRNA CNTL) and shRNA targeting mouse SIRT1 (LV-PGK-EGFP-H1-shRNA SIRT1) were produced in HEK293T cell line with a four-plasmid system, as previously described^49^. Lentiviral particles were re-suspended in 1% BSA in sterile PBS. The viral particle content of batches was evaluated by assessing HIV-1 p24 antigen levels by ELISA (Retro Tek, Gentaur, Paris, France). Concentrated viral stocks were stored at −80 °C until use.

### Stereotaxic injection into the striatum

Five-week-old mice were anaesthetized with avertin (14 μl g^−1^ and 250 mg kg^−1^ intraperitoneally). Four hundred nanograms of p24 antigen of lentiviral vectors in a final volume of 2.5 μl encoding for the specific transgenes depending on the experiment were stereotaxically co-injected into the striatum in the following coordinates: anteroposterior: +0.6 mm, lateral: ±1.8 mm, ventral:−3.3 mm and tooth bar: 0.

### Behaviour assays

Six-week-old mice were submitted to a battery of motor tests starting 2 days before the beginning of the study (*t*=0) and every 2/3 weeks until 8/9 weeks, depending on the study. All tests were performed in a dark room with, at least, 60 min of acclimatization to the experimental room and at the same period of the day (during the morning until early afternoon). Details of each test can be found in the Supplementary Methods.

### Histology

We performed fluorescent and colorimetric immunohistochemistry on serial coronal and saggital sections spanning rostrocaudal extent of the striatum or mediolateral extent of the cerebellum. All equivalent sections between the groups were evaluated and treated/imaged under the same conditions. Details of the procedure and of the quantifications can be found in the Supplementary Methods.

### Immunoblotting analysis

Immunoblots were performed as described^13^,^16^ and specific details can be found in the Supplementary Methods. Images have been cropped for presentation. Full-size images are presented in Supplementary Figs 6–17.

### Gene expression

Total RNA were isolated using commercially available kit (Macherey-Nagel) according to the manufacturer's instructions. Complementary DNA was then obtained by conversion of total RNA with iScript Selected cDNA Synthesis kit (Bio-Rad) according to manufacturer's instructions and stored at −20 °C. Gene expression was determined by quantitative PCR as described^15^. See Supplementary Table 1 and Supplementary Methods for more details.

### Engineering of shRNA

An shRNA negative (control), an shRNA targeting SIRT1 and an shRNA targeting ATG5 were created. For each one, a pair of oligonucleotides was designed. The sequences of each pair of oligonucleotides can be found in the Supplementary Experimental Procedures. Each pair of oligonucleotides were annealed and inserted in linearized p-ENTR/pSUPER+ (AddGene 575-1). The H1-shRNA cassette was then transferred, with LR clonase recombination system, into SIN-cPPT-PGK-EGFP-WHV-LTR gateway destination vector. See Supplementary Table 1 for more details.

### Neuroblastoma cell culture

Mouse neuroblastoma cell line (Neuro-2a cells) was obtained from the American Type Culture Collection cell biology bank (CCL-131) and maintained in DMEM medium supplemented with 10% fetal bovine serum, 100 U ml^−1^ penicillin and 100 mg ml^−1^ streptomycin (Gibco) (complete medium) at 37 °C in 5% CO_2_/air atmosphere. Neuro2a cell infection was performed as previously described^16^. At 2 weeks post infection, cells were incubated, or not with 100 μM of chloroquine (Sigma-Aldrich) during 3 h and then were lysed for western blot processing. In shRNA targeting ATG5/SIRT1 study, Neuro2a cells were plated and 24 h later were transduced with respective plasmids, using polyethyleneimine as transfection reagent. Forty-eight hours later, cells were collected and lysed for western blot processing.

### Statistical analysis

Statistical analysis was performed with paired or unpaired Student's *t*-test and one-way or two-way analysis of variance followed by the adequate *post hoc* test, for multiple comparisons. Results are expressed as mean±s.e.m. Significant thresholds were set at *P*<0.05, *P*<0.01 and *P*<0.001, as defined in the text.

### Additional methods

Detailed methods, including behavioural tests, histological processing and biochemical analysis are described in Supplementary Methods.

## Additional information

**How to cite this article:** Cunha-Santos, J. *et al*. Caloric restriction blocks neuropathology and motor deficits in Machado–Joseph disease mouse models through SIRT1 pathway. *Nat. Commun.* 7:11445 doi: 10.1038/ncomms11445 (2016).

## Supplementary Material

Supplementary Information Supplementary Figures 1-17, Supplementary Table 1, Supplementary Methods and Supplementary References.
