# Supplementary material for: Caloric restriction blocks neuropathology and motor deficits in Machado–Joseph disease mouse models through SIRT1 pathway
Source: Nat Commun. 2016 May 11;7:11445. doi: 10.1038/ncomms11445 (PMC4865854; doi:10.1038/ncomms11445)
Supplement: Supplementary Information — Supplementary Figures 1-17, Supplementary Table 1, Supplementary Methods and Supplementary References. [file ncomms11445-s1.pdf]

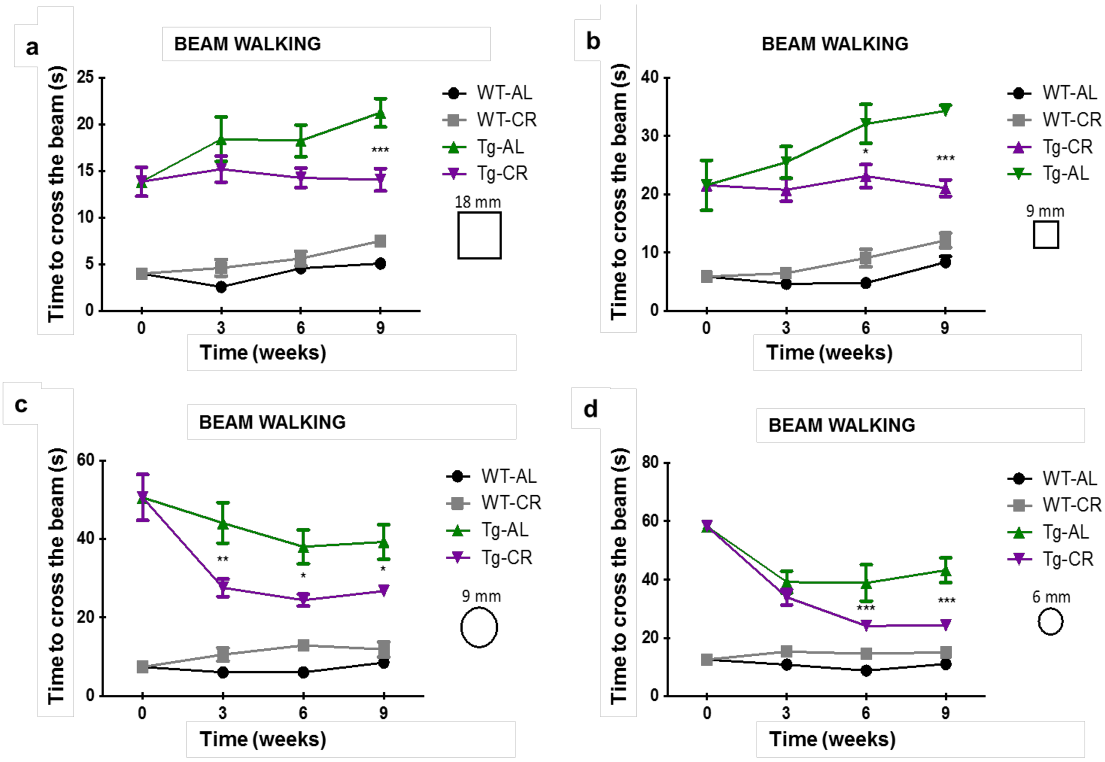

Supplementary Figure 1. Caloric restriction alleviates MJD motor incoordination. (a-d) Beam walking test in four different beams, with different diameter and shape, during the experimental time course. Motor performance reached significance for the 9 mm beams and 6 mm round beam six weeks after the beginning of the study.

Data represent mean ± SEM. n.s. p>0.05; *p<0.05; **p<0.01; ***p<0.001 compared to Tg-AL. **(a-d)** 2-way ANOVA with Bonferroni's post-hoc test. WT-AL n=5; WT-CR n=7; Tg-AL n=6; Tg-CR n=7.

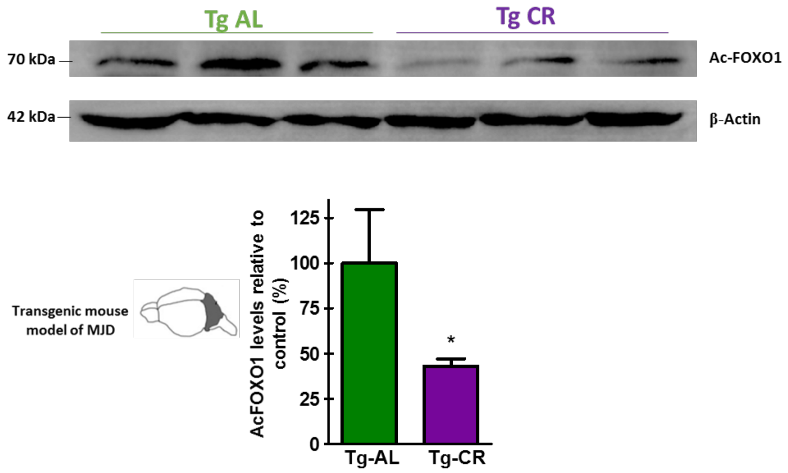

Supplementary Figure 2. Caloric restriction increases SIRT1 levels and activity in transgenic MJD mice. Cerebella of transgenic MJD mice under CR (Tg-CR) present lower levels of acetylated FOXO1a, in comparison with transgenic *ad libitum* fed animals, suggesting that SIRT1 activity is increased by CR. Data represent mean ± SEM. *p<0.05. Compared to Tg-AL. Unpaired Student's t-test. n=3.

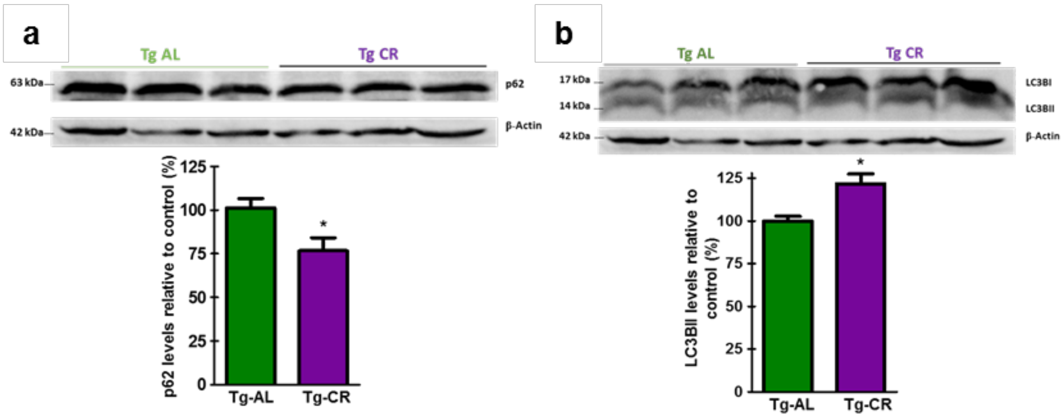

Supplementary Figure 3. Caloric restriction (CR) increases SIRT1 levels and activity in transgenic MJD mice. (a) Cerebella of caloric restricted transgenic MJD mice (Tg-CR) exhibited a significant higher LC3BII levels. **(b)** Cerebella of Tg-CR exhibited lower p62 levels, in comparison with transgenic *ad libitum* fed animals (Tg-AL). Data represent mean ± SEM. *p<0.05. Relative to Tg-AL.**(a,b)** Unpaired Student's t-test. n=3.

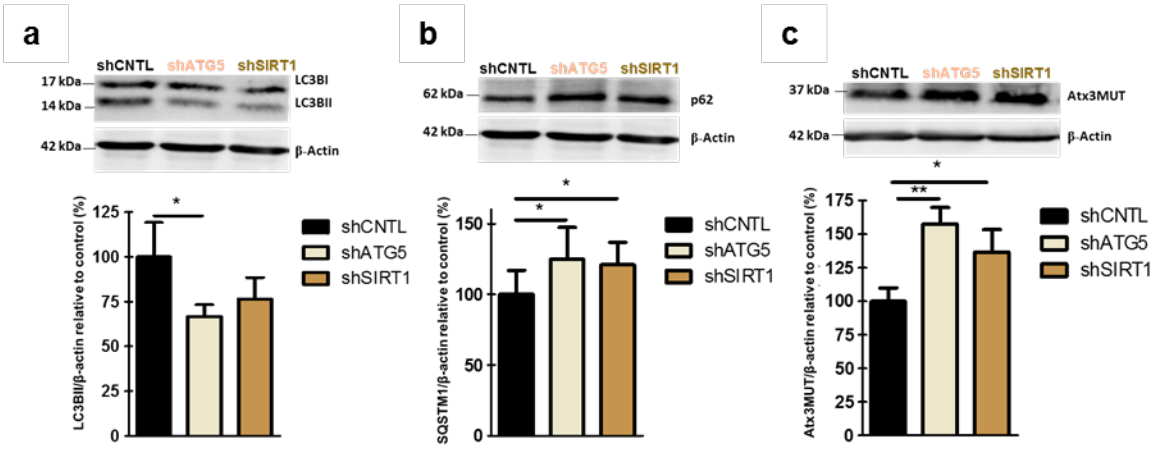

Supplementary Figure 4. ATG5 or SIRT1 knockdown induces a disruption of autophagic flux and an accumulation of mutant ataxin-3 in Neuro2a cells expressing mutant ataxin-3. A.B. Autophagy is disrupted with ATG5 or SIRT1 genetic silencing autophagy. **(a)** LC3BII levels are decreased with the knockdown of ATG5 or SIRT1. **(b)** p62 levels are increased when ATG5 or SIRT1 are genetically silenced. **(c)** Autophagy disruption promoted by ATG5 or SIRT1 knockdown induces mutant ataxin-3 accumulation, suggesting that mutant ataxin-3 is eliminated by autophagy. Data represent mean ± SEM. *p<0.05; **p<0.01. Relative to shCNTL. **(a-c)** Paired Student's t-test. n=3.

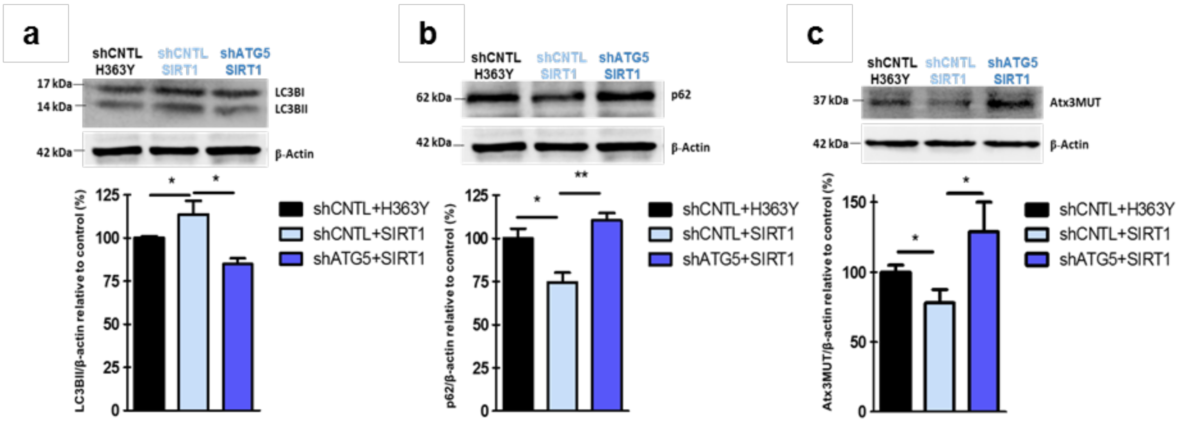

Supplementary Figure 5. SIRT1 overexpression induces autophagic flux and increases mutant ataxin-3 clearance and these effects are reverted with ATG5 knockdown, in Neuro2a cells expressing mutant ataxin-3. (a,b) Autophagy is activated with SIRT1 overexpression and blocked upon ATG5 knockdown. **(a)** SIRT1 overexpression induces the increase in LC3BII levels and with the knockdown of ATG5 this effect is blocked. **(b)** p62 levels are decreased with SIRT1 overexpression in the absence of ATG5 knockdown and increased when ATG5 is genetically silenced, in comparison with cells transduced with H363Y and a control shRNA. **(c)** SIRT1 overexpression induces mutant ataxin-3 clearance although when autophagy is disrupted, promoted by ATG5 knockdown, mutant ataxin-3 is accumulated. Data represent mean ± SEM. *p<0.05; **p<0.01. Relative to shCNTL. **(a-c)** Paired Student's t-test. n=3.

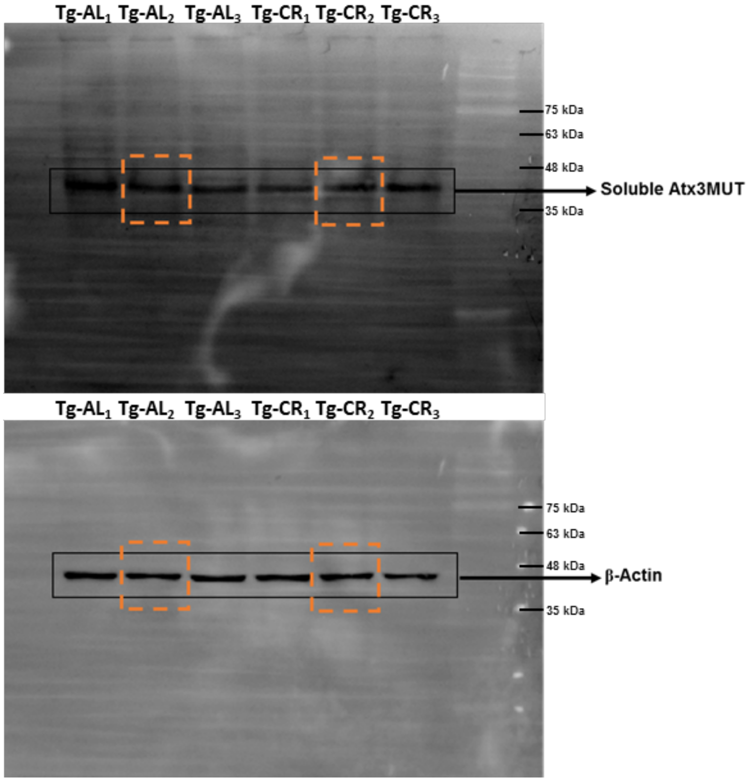

Supplementary Figure 6. Uncropped blots for Fig. 2f. Note that orange dashed represents the cropped image.

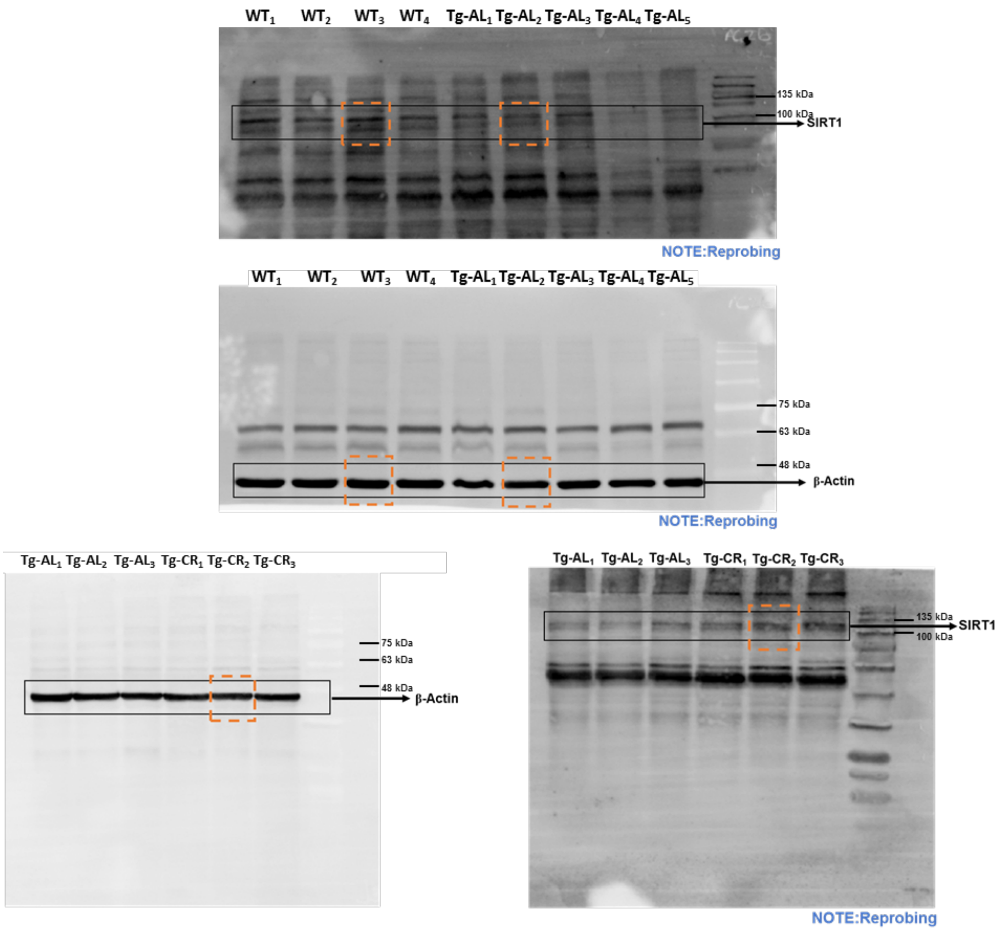

Supplementary Figure 7. Uncropped blots for Fig. 3b. Note that orange dashed represents the cropped image.

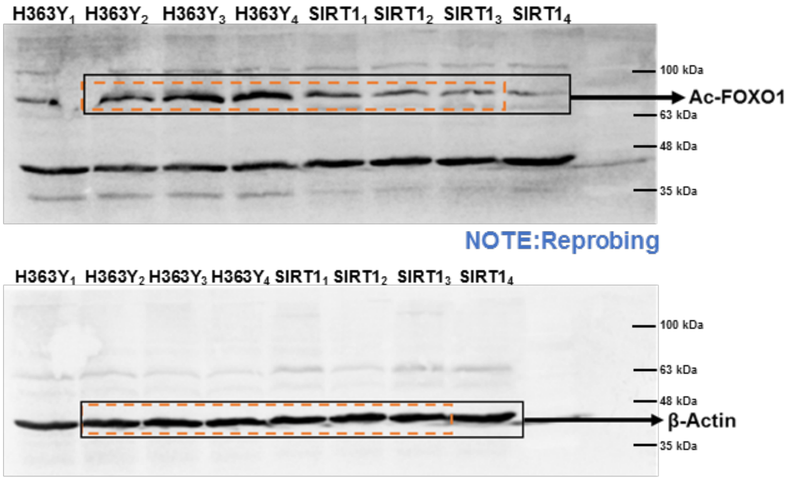

Supplementary Figure 8. Uncropped blots for Fig. 4c. Note that orange dashed represents the cropped image.

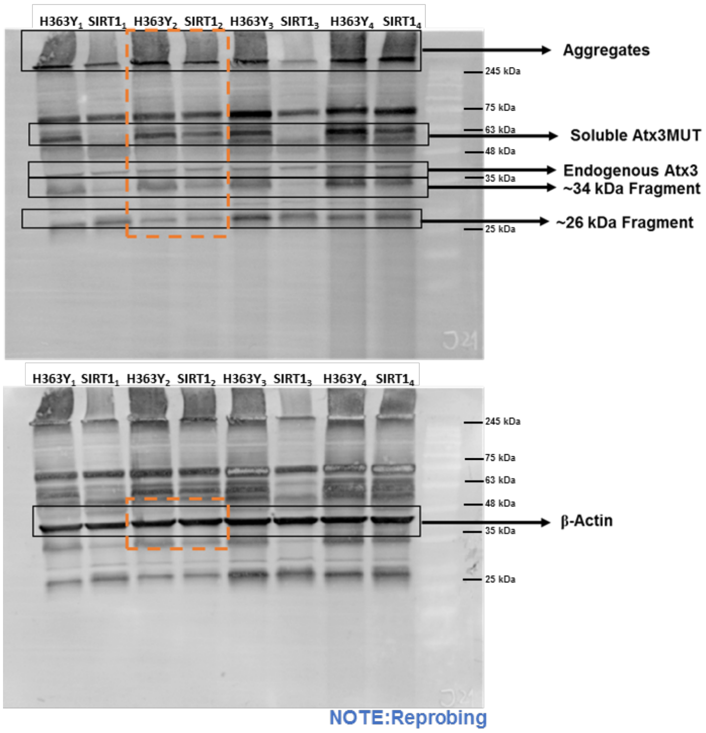

Supplementary Figure 9. Uncropped blots for Fig. 4f. Note that orange dashed represents the cropped image.

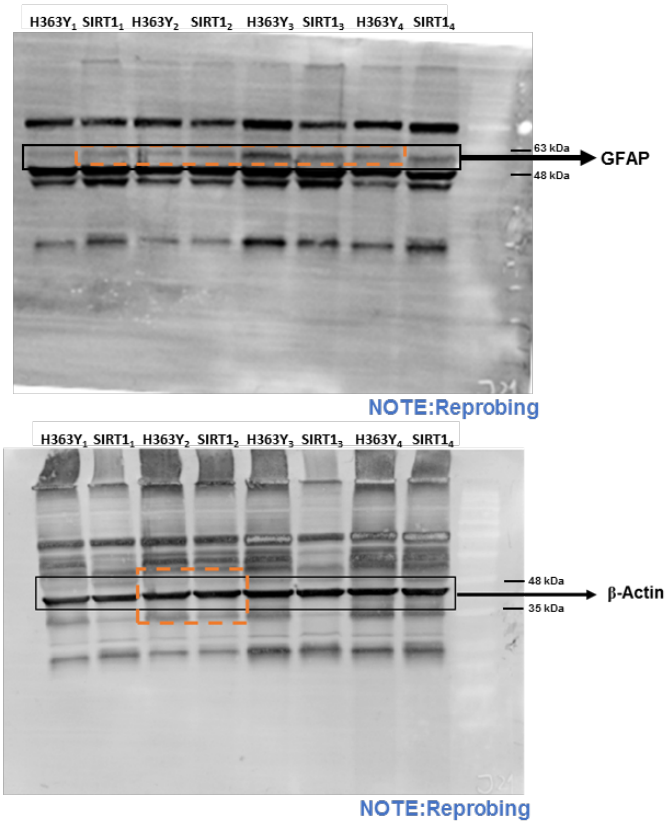

Supplementary Figure 10. Uncropped blots for Fig. 5c. Note that orange dashed represents the cropped image.

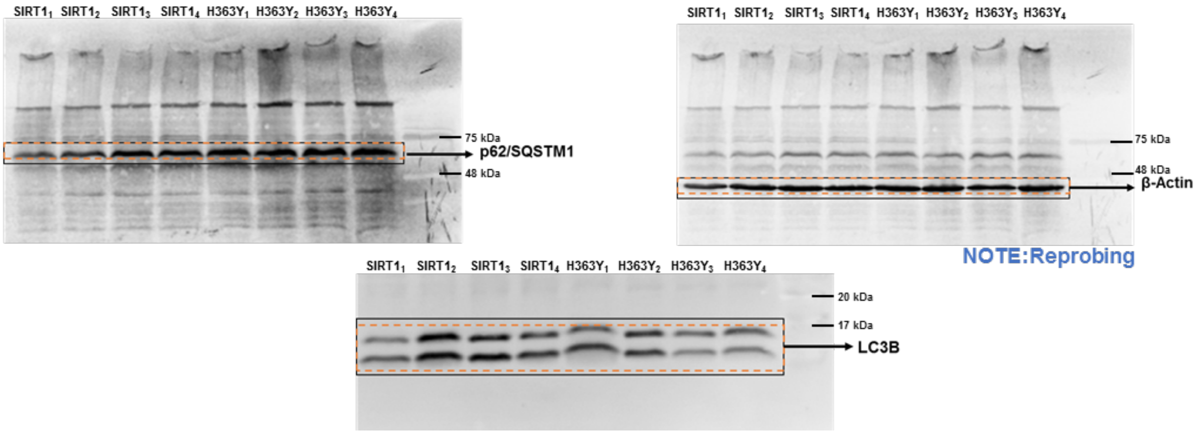

Supplementary Figure 11. Uncropped blots for Fig. 5j,k. Note that orange dashed represents the cropped image.

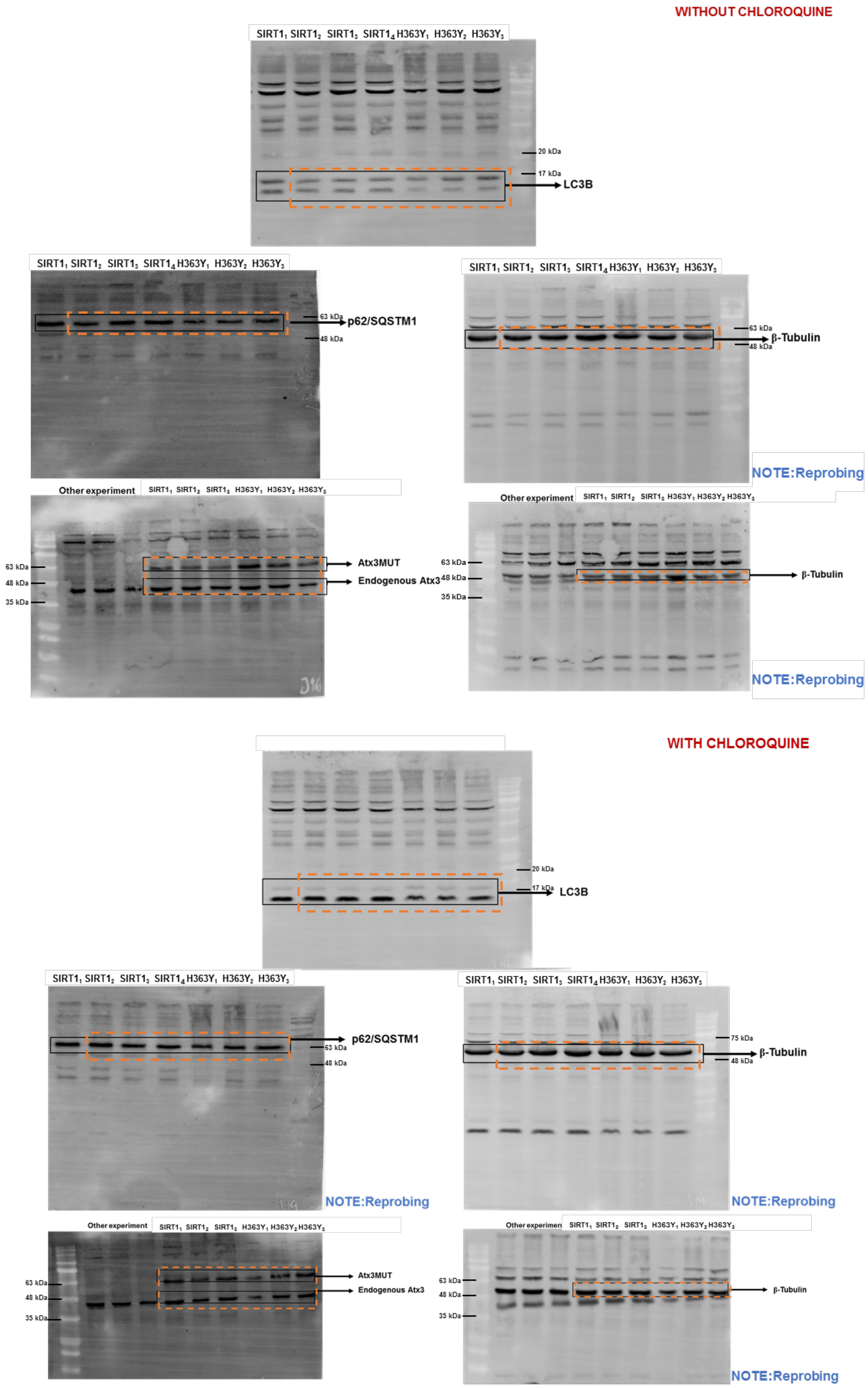

Supplementary Figure 12. Uncropped blots for Fig. 5l-n. Note that orange dashed represents the cropped image.

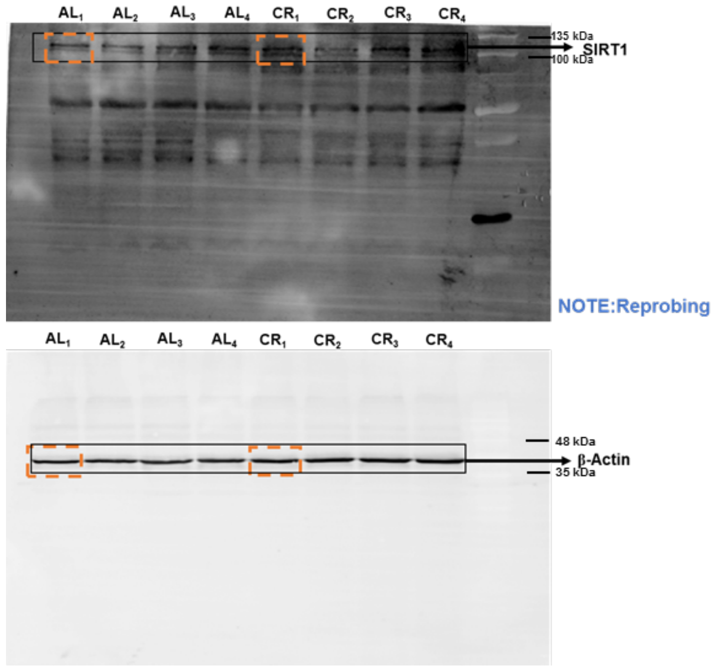

Supplementary Figure 13. Uncropped blots for Fig. 6e. Note that orange dashed represents the cropped image.

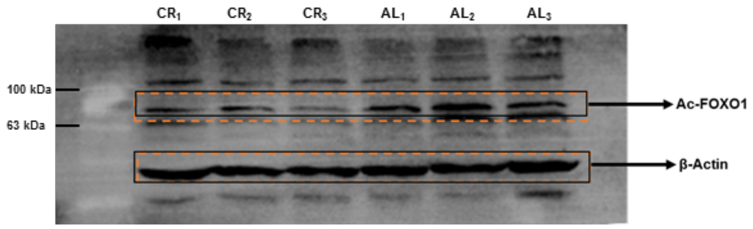

Supplementary Figure 14. Uncropped blots for Fig. S2. Note that orange dashed represents the cropped image.

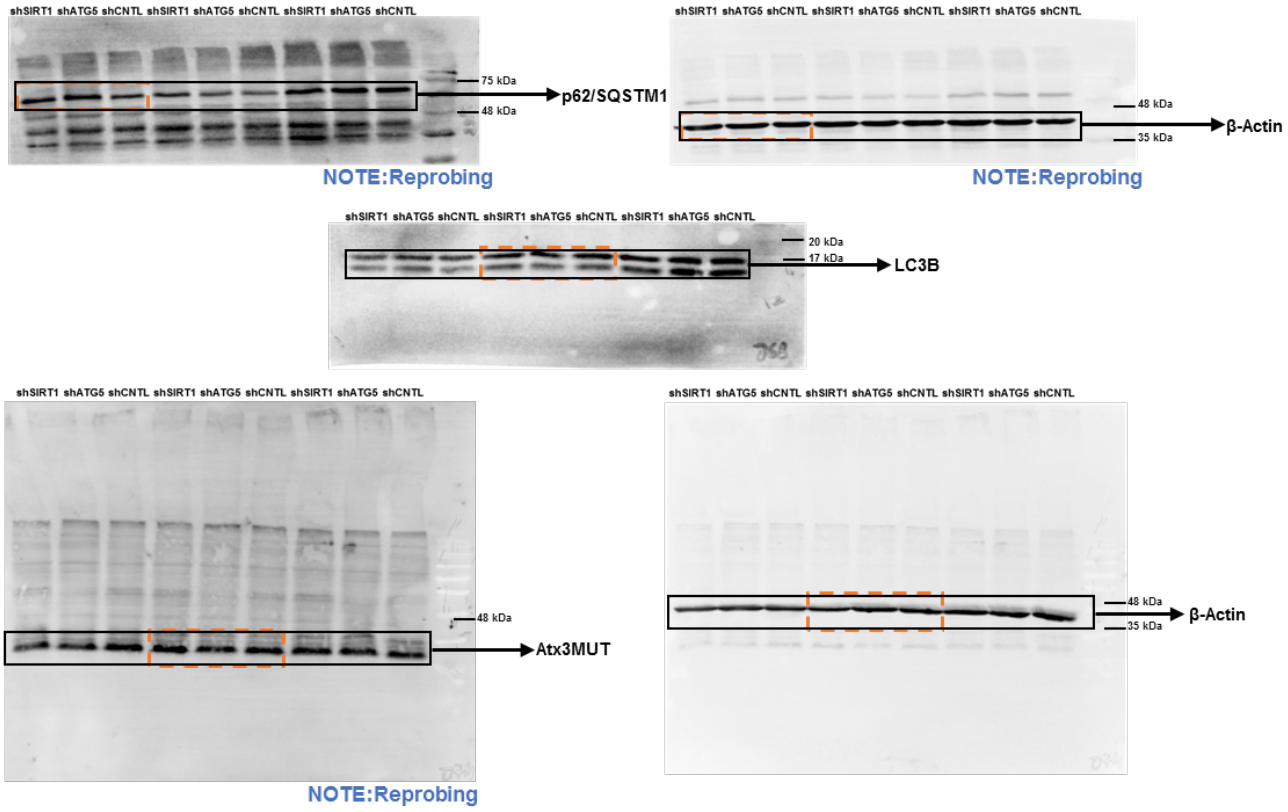

Supplementary Figure 15. Uncropped blots for Fig. S3. Note that orange dashed represents the cropped image.

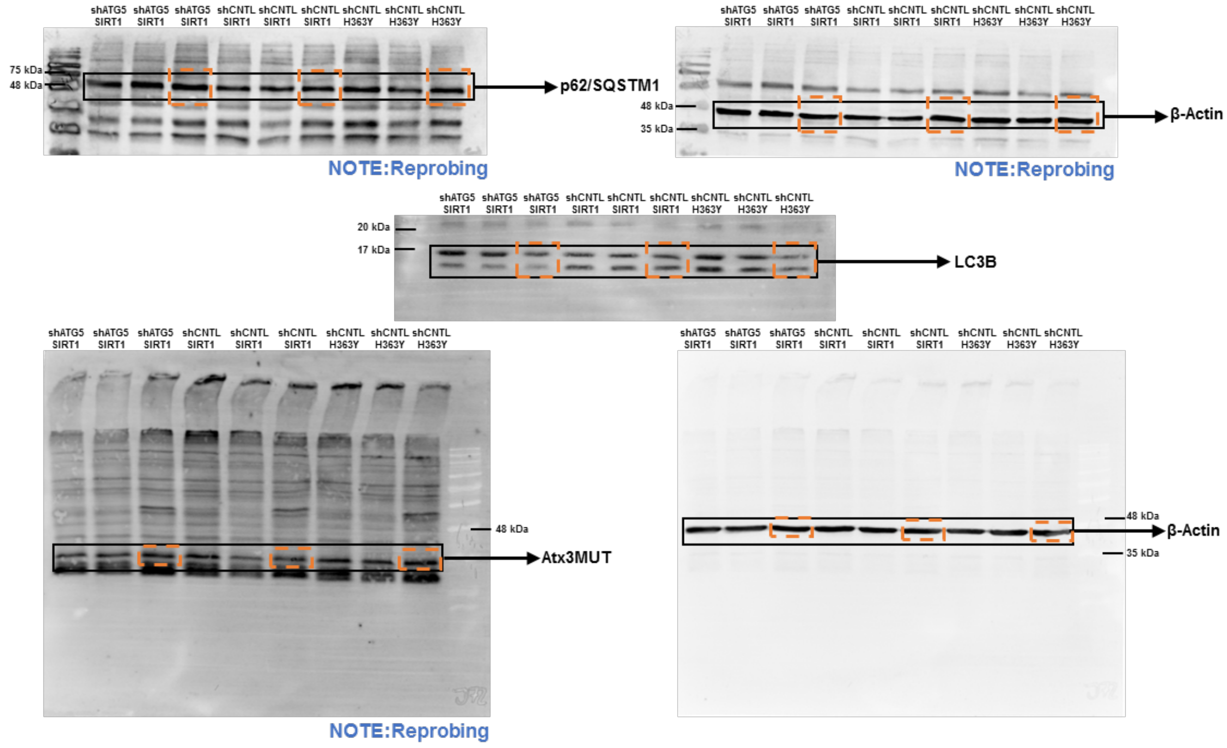

Supplementary Figure 16. Uncropped blots for Fig. S4. Note that orange dashed represents the cropped image.

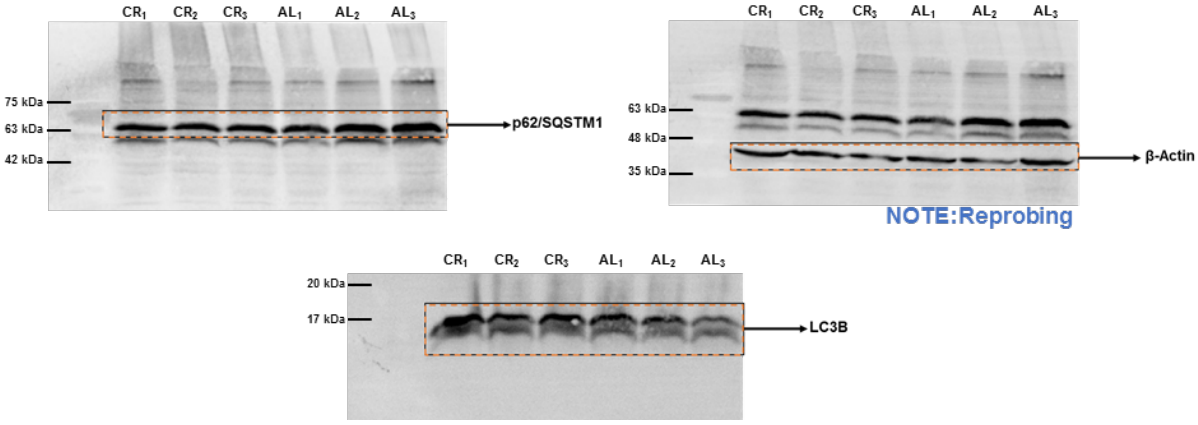

Supplementary Figure 17. Uncropped blots for Fig. S5. Note that orange dashed represents the cropped image.

Supplementary Table 1

**OLIGOS**	**SEQUENCE**
hSIRT1 F	5'TGCTCGCCTTGCTGTAGACTTC3'
hSIRT1 R	5'GGCTATGAATTTGTGACAGAGAGATGG3'
hGAPDH F	5'TGTTCGACAGTCAGCCGCATCTTC3'
hGAPDH F	5'CAGAGTTAAAAGCAGCCCTGGTGAC3'
shRNA Neg top	5'GATCCCCCAACAAGATAAGAGCACCAATTCAAGAGATTGGTGCTC TTCATCTTGTTG3TTTTTA3'
shRNA Neg bot	5'AGCTTAAAAACAACAAGATGAAGAGCACCAATCTCTTGAATTGGTG CTCTTCATCTTGTTGGGG3'
shRNA SIRT1 top	5'GATCCCCGCCATGTTTGATATTGAGTATTTCAAGAGAATACTCAAT ATCAAACATGGC3TTTTTA'
shRNA SIRT1 bot	5'AGCTTAAAAAGCCATGTTTGATATTGAGTATTCTCTTGAAATACTCA ATATCAAACATGGCGGG3'
shRNA ATG5 top	5'GATCCCCAGCCGAAGCCTTTGCTCAATGTTCAAGAGACATTGAGC AAAGGCTTCGGCTTTTTA3'
shRNA ATG5 bot	5'AGCTTAAAAAAGCCGAAGCCTTTGCTCAATGTCTCTTGAACATTGA GCAAAGGCTTCGGCTGGG3'

Supplementary Methods

Behavioral Test - Stationary and Accelerated Rotarod: Rotarod apparatus Letica Scientific Instruments, model LE 8200 (Panlab, Barcelona, Spain) was used. Rotarod tests were performed as we previously described[1]. In accelerated rotarod test, mice were placed at accelerated speed (accelerating speed from 4 r.p.m. to 40 r.p.m. over a period of 5 minutes). On stationary rotarod, mice were placed on the rotarod at a constant velocity (5 r.p.m. for a maximum of 5 minutes). The time during which mice remain running in the rotation roll was recorded. For each test and time point, each animal performed two sets of two trials with a 20-min inter-trial interval.

Vertical Pole Test: The vertical pole test was used to assess motor coordination and balance of mice[2]. Each mouse was positioned head-upward on the top of a round rough-surfaced pole (with 52 cm of height and 1 cm of diameter). The vertical pole was initially positioned horizontally and then slowly inclined to 90º. The time to orient downward (t-turn) and the time to reach the floor (t-descend) was recorded and the maximum observation time defined was 2 min. Five consecutive trials were performed with an inter-trial interval of 60 s.

Swimming Test: Mice were placed in the border of a rectangular aquarium (length: 70 cm; height: 20 cm; width: 15 cm), with a platform located at 9 cm upper the floor (length: 7cm; width: 6 cm) and in the opposite side. The aquarium was filled up to the area of the platform with water at 24-26ºC. The time that mice took to reach the platform was recorded. The time of four trials with an inter-trial interval of 60 seconds were recorded. The first trial was considered as an exploratory trial and the results were expressed as the average of the other three trials.

Beam walking test: In order to evaluate motor coordination and balance of mice, the ability of the mice to cross a graded series of narrow beams to reach an enclosed escape platform, was evaluated[3]. The test was performed in four long wood beams (1 m): two round beams with 9 or 6 mm diameter and two square beams with 18 or 9 mm square wide. The tests were performed sequentially from the widest to the narrowest beam: the first beam was the 18 mm square beam, followed by the 9 mm square beam and the 9 mm round beam, ending with the 6 mm round beam. Each beam was placed horizontally, 25 cm above the bench surface, with one end attached on a narrow support and the other end fixed to a bounded box (20 cm square) into which the mouse could escape. Mice were allowed up to 60 sec to transverse each beam, and the time was recorded. Any animal that did not cross in 60 sec was assigned the maximum value of 60 sec for analysis.

Footprint pattern: Different parameters of gait can be evaluated simply and effectively tracking the footprint pattern. This test was used to evaluate the effect of caloric restriction in the gait of mice and was performed as we previously described[1]. To obtain footprints, mice feet were paint with black and white non-toxic paints, respectively in the hind and forefeet. Then, animals were allowed to walk along 100 cm long, in a 10 cm wide runaway fresh sheet of green paper, in an apparatus with 15 cm high walls. A fresh sheet of green paper was replaced on the floor for each new mouse. In order to analyze footprint patterns some parameters were explored (all measured in

centimeters). Stride length, the average distance of forward movement between each stride, was measured. This parameter was determined by the measurement of the perpendicular distance of a given step to a line connecting its opposite preceding and proceeding steps. The average of three strides was obtained. To explore uniformity of step alternation the distance from left or right front footprint/hind footprint overlap was measured. A perfect overlap, recorded as zero, was considered when the centre of the hind footprint fell on the top of the centre of the preceding front footprint. When the footprint did not overlap, the distance between the centre of footprints was verified. A sequence of four consecutive steps was chosen for evaluation, excluding footprints made at the beginning and the end of the run, where the animal was initiating or finishing the movement, respectively.

Open Field Test: Open-field test was used to explore locomotor horizontal activity and anxiety-like behaviors of mice. Mice were placed in the center of a 50x50 cm arena with 50 cm high walls and movement activity was recorded for 40 min with Acti-Track System (PanLab, Barcelona, Spain). In order to reduce any type of novelty it was guaranteed that cage changes were performed at least 24 hours prior to test. All the experiments were performed by the same operator and during the experiment the operator was outside the experimental room. Total distance travelled in the experimental time, mean velocity of mice and time and distance travelled in the center of arena was recorded.

Tissue preparation for Histological processing : Mice were sacrificed with an overdose of avertin (2.5 times of 14 μl/g, 250 mg/kg, intraperitoneally). Transcardial perfusion with phosphate solution and fixation with 4% paraformaldehyde were performed. The brain was collected and post-fixed in 4% paraformaldehyde for 24 hours and cryoprotected by incubation in 25% sucrose/phosphate buffer. After that, dry brains were frozen at -80ºC and 25 μm coronal sections from injected mice or 35 μm saggital sections from transgenic MJD and littermate mice were cut using a cryostat (LEICA CM3050S, Leica Microsystems) at -21ºC.

Immunohistochemistry: After the blockage of endogenous peroxidases with phenylhidrazyne/phosphate solution and incubation in PBS/0.1% Triton X-100 with 10% normal goat serum (Gibco), free-floating sections were overnight incubated at 4ºC in blocking solution with primary antibodies: mouse monoclonal anti-ataxin 3 antibody (1H9; 1:5000; #5360 Merck Millipore), rabbit anti-dopamine and cyclic AMP-regulated neuronal phosphoprotein 32 (DARPP-32) antibody (1:1000; AB#10518 Merck Millipore); followed by incubation with respective biotinylated secondary goat anti-mouse or anti-rabbit antibodies (1:200; Vector Laboratoires). Bound antibodies were visualized using the VECTASTAIN® ABC kit, with 3',3'-diaminobenzidine tetrahydrocloride (DAB metal concentrate; Pierce) as substrate. Dry sections were mounted in gelatin-coated slides, dehydrated with ethanol solutions and xylene and mounted in Eukit (Sigma-Aldrich).

Free-floating sections were incubated in PBS/0,1% Triton X-100 containing 10% normal goat serum (Gibco), and then incubated overnight at 4ºC in blocking solution with primary antibodies:

mouse polyclonal anti-glial fibrillary acidic protein (GFAP) antibody (1:1000; Z0334 Dako, Glostrup, Denmark); rabbit anti-ionized calcium binding adaptor molecule 1 (Iba-1) antibody (1:1000; #019-19741 Wako Chemicals, USA); rabbit anti-sirtuin 1 antibody (1:250; #2028 Cell Signaling Technology); mouse monoclonal anti-HA antibody (1:1000; ab-hatag InvivoGen, San Diego, CA, USA). Sections were washed and incubated for 2h at room temperature with the corresponding secondary antibodies coupled to fluophores goat anti-mouse/goat anti-rabbit Alexa Fluor 488 or Alexa Fluor 594 (1:250, Molecular Probes-Invitrogen, Eugene, OR) diluted in the respective blocking solution. The sections were washed and incubated during 10 min with 4',6'-diamidino-2-phenylindole DAPI (Sigma; St. Louis; MO), washed and mounted in mowiol on microscope slides.

Immunoreactivity of mouse sections was analyzed as previously described[4]. Staining was visualized with Zeiss Axioskop 2 plus or Zeiss Axiovert 200 imaging microscopes (Carl Zeiss MicroImaging, Oberkochen, Germany) equipped with AxioCam HR color digital cameras (Carl Zeiss Microimaging) and 35, 320, 340 and 363 Plan-Neofluor or 363 Plan/Apochromat objectives using the AxioVision 4.7 software package (Carl Zeiss Microimaging). Quantitative analysis of fluorescence was performed with a semiautomated image-analysis software package and images were taken under the same image acquisition conditions and uniform adjustments of brightness and contrast were made to all images (ImageJ; NIH; Bethesda, MD).

Cresyl Violet Staining: Dry pre-mounted sections were stained with cresyl violet solution for 1 min, differentiated in 70% ethanol, dehydrated twice by passing through 95% ethanol, 100% ethanol and xylene solutions and mounted onto microscope slides with Eukit (Sigma).

Analysis of the volume of DARPP-32 depletion region: The extent of striatal mutant ataxin-3 72Q lesions was evaluated by photographing, with a x5 objective, 12 sections stained with DARPP-32 per animal (25 µm-thick sections at 200 µm intervals) were selected to obtain rostrocaudal sampling of the striatum. The area of the lesion was quantified with a semi-automated image-analysis software package (ImageJ, NIH, USA). The area of the striatum showing a loss of DARPP-32 staining was measured for each animal, with an operator-independent macro. The volume was then estimated with the following formula: volume = $d(a_1+a_2+\ldots+a_{11}+a_{12})$, where d is the distance between serial sections (200 µm) and $a_{1\text{-}12}$ are DARPP-32 depleted areas for individual serial sections[5] The depleted area corresponds to area with a gray-scale value lower than the mean gray-scale value of all pixels measured in the lesioned area.

Cell counts of ataxin-3 inclusions: Coronal sections showing entire striatum (12 sections *per* animal) were scanned with x20 objective. The analyzed areas of the striatum encompassed the entire region containing ataxin-3 inclusions, as revealed by staining with anti-ataxin-3 antibody. All inclusions were blindly manually counted using a semiautomated image analysis software package (Image J, NIH, USA).

Pyknotic nuclei counting: The number of pyknotic nuclei in the area under the needle tract was evaluated. Specifically eight regions in the transduced region under the needle tract of each

injection were photographed, with a x40 objective. The total number of the pyknotic nuclei in this area was blindly counted for each animal and each hemisphere using a semiautomated image analysis software (Imaje J, NIH, USA).

Quantitative analysis of haemagglutinin-tagged (HA) aggregates: Quantification of haemagglutinin-tagged ataxin-3 positive inclusions was performed after a HA immunohistochemistry and imaging of eight sagittal sections with 35 µm-thick spread over the lateral extent of cerebellum of each animal, using a x20 objective on a Zeiss Axioskop 2 plus imaging microscope. The number of HA inclusions in Purkinje cells were blindly counted. The number of inclusions in Purkinje cells in the cerebellum was estimated by the following formula: total number = $2s(n_1+n_2+n_3+\ldots+n_8)$, where s represents the number of intermediate sections (8), n_{1-8} represents the number of aggregates present in each section and this was multiplied by 2 to estimate the total number in the two hemispheres.

Quantification of granular and molecular layers size and cerebellar volume: Quantification of granular and molecular layers size and cerebellar volume was made using 8 cresyl violet staining sagittal sections with 35 µm-thick spread over lateral extent of cerebellum in a blind fashion. For each section, lobule V and lobule IX cerebellar cortex were digitalized in x20 objective. For each acquired field, four measurements were made blindly in the same region for all animals. Results were converted to µm using Image J software (NIH, USA). The showed results are the average of the thickness of each layer in lobule V and IX together. For cerebellar volume evaluation, x5 objective images were obtained, of each section and was blindly evaluated the area of cerebellum using Image J software (NIH, USA). Cerebellar volume was estimated using the following formula: $2d(a_1+a_2+\ldots+a_8)$, where d represents the distance between two followed sections (280 µm), a_{1-8} represents the calculated area of each section and this was multiplied by two to estimate the volume of whole cerebellum.

Tissue preparation for Immunoblot Procedure: Mice were sacrificed by a lethal dose of avertin. Cerebellum or striatal dissection were performed and tissues were lysated with radioimmunoprecipitation assay-buffer solution (RIPA buffer; 50 mM Tris-HCl pH=8; 150 nM NaCl; 1% NP-40 nonyl phenoxypolyethoxylethanol); 0.5% sodium deoxycholate; 0.1% sodium dodecyl sulphate) containing protease inhibitors (Roche Diagnostics GmbH) and supplemented with 1 mM PMSF (phenylmethylsulphonyl fluoride, Sigma-Aldrich) and 10 µg/mL DTT (dithiothreitol, Sigma-Aldrich). Tissue lysates were prepared by 2 series of 4 sec ultra-sound pulse (1 pulse/sec). *In vitro* samples were obtained scrapping cells with supplemented RIPA buffer and collecting cellular lysates, followed by centrifugation at 12.000 r.p.m. during 10 minutes at 4ªC. Total protein lysates were stored at -80ºC and protein concentration was determined with BCA protein assay (Pierce Biotechnology, Thermo Scientific, USA).

Western Blot procedure: Samples were previously denatured with 2x sample buffer (10% β-mercaptoethanol, 4% sodium dodecyl sulphate (SDS), 0.25 M Tris-HCl, 8M urea) and incubated during 5 min at 95ºC. Equal amounts of protein (50 µg or 25 µg) were resolved on 12% SDS-

PAGE and transferred onto polyvinylidene fluoride (PVDF) membranes (GE Healthcare, United Kingdom) according to standard protocols. The membranes were blocked by incubation in 5% non-fat milk powder in 0.1% Tween 20 in Tris buffered saline (TBS-T), and incubated overnight at 4°C with primary antibody: mouse monoclonal anti-ataxin-3 antibody (1H9; 1:3000; #5360 Merck Millipore); rabbit anti-sirtuin 1 antibody (1:1500; #2028 Cell Signaling Technology); rabbit monoclonal anti-p62 antibody (1:1000; #5117 Cell Signaling Technology); rabbit monoclonal anti-LC3B antibody (1:1000; #2775 Cell Signaling Technology); mouse anti-polyclonal glial fibrillary acidic protein (GFAP) antibody (1:1000; #3670 Cell Signaling Technology); rabbit anti-AcFOXO1 antibody (1:1000; sc-49437 Santa Cruz Biotech); mouse anti-β-actin antibody (clone AC74; 1:5000; A5316 Sigma-Aldrich); mouse anti-HA antibody (1:1500; ab-hatag InvivoGen, San Diego, CA, USA); mouse anti-β-tubulin antibody (1:10.000; T7816 Sigma-Aldrich), followed by the incubation with the corresponding alkaline phosphatase-linked secondary goat anti-mouse or anti-rabbit antibody. Bands were visualized with enhanced chemifluorescence substrate (ECF, GE Healthcare) and chemiflourescence imaging (VersaDoc Imaging System Model 3000, BioRad). Semi-quantitative analysis was carried out based on the optical density of scanned membranes (Quantity One; 1-D image analysis software version 4.6.6; Bio-Rad). The specific optical density was then normalized with respect to the amount of β-actin or β -tubulin loaded in the corresponding lane of the same gel.

Isolation of total RNA from mouse tissues and cDNA synthesis: Animals were sacrificed with a lethal dose of avertin. Cerebellum or striatum of mice were dissected and stored in tubes containing RNA*later* RNA stabilization reagent (QIAGEN) and were kept at -80ºC until RNA isolation. Total RNA was isolated with NucleoSpin RNA isolation kit (Macherey-Nagel) according to manufacturer's instructions and was preceded by homogenization of the tissue with Qiazol reagent (Qiagen) and d-clorophorm and a treatment with DNase I (Macherey-Nagel) was performed. Total amount of RNA was quantified by optical density (OD) using a Nanodrop 2000 Spectrophotometer (Thermo Scientific) and RNA was stored at -80ºC. cDNA was then obtained by conversion of total RNA with iScript Selected cDNA Synthesis kit (Bio-Rad) according to manufacturer's instructions and stored at -20ºC.

Quantitative real time polymerase-chain reaction: qPCR was performed in the StepOne Plus Real-Time PCR System (Applied Biosystems) using 96-well microliter plates and the SsoAdvanced SYBR Green Supermix (Bio-Rad). qRT-PCR was carried out in 10 µL reaction volume. Primers for mouse SIRT1, IL-6, IL-1β, TNF-α, IL-10 and GADPH were pre-designed and validated by QIAGEN (QuantiTect Primers, QIAGEN). Primers for human SIRT1 and human GADPH were designed using PrimerBlast Software and the sequences are listed below (TABLE S1). Appropriate negative controls were also prepared. All reactions were performed in duplicate and according to the manufacturer's recommendations: 95ºC for 30 sec, followed by 45 cycles at 95ºC for 5 sec and 60ºC for 30 sec. The amplification rate for each target was evaluated from the cycle threshold (Ct) numbers obtained with cDNA dilutions, with correction for GADPH levels. The mRNA fold increase or fold decrease with respect to control samples was determined by the Pfäffl method.

Supplementary References

1. Nobrega, C., *et al.* Overexpression of mutant ataxin-3 in mouse cerebellum induces ataxia and cerebellar neuropathology. *Cerebellum* **12**, 441-455 (2013).
2. Matsuura, K., Kabuto, H., Makino, H. & Ogawa, N. Pole test is a useful method for evaluating the mouse movement disorder caused by striatal dopamine depletion. *J Neurosci Methods* **73**, 45-48 (1997).
3. Simoes, A.T., Goncalves, N., Nobre, R.J., Duarte, C.B. & Pereira de Almeida, L. Calpain inhibition reduces ataxin-3 cleavage alleviating neuropathology and motor impairments in mouse models of Machado-Joseph disease. *Hum Mol Genet* (2014).
4. Goncalves, N., Simoes, A.T., Cunha, R.A. & de Almeida, L.P. Caffeine and adenosine A(2A) receptor inactivation decrease striatal neuropathology in a lentiviral-based model of Machado-Joseph disease. *Ann Neurol* **73**, 655-666 (2013).
5. de Almeida, L.P., Ross, C.A., Zala, D., Aebischer, P. & Deglon, N. Lentiviral-mediated delivery of mutant huntingtin in the striatum of rats induces a selective neuropathology modulated by polyglutamine repeat size, huntingtin expression levels, and protein length. *J Neurosci* **22**, 3473-3483 (2002).
